# Structural determinants for ERK5 (MAPK7) and leucine rich repeat kinase 2 activities of benzo[*e*]pyrimido-[5,4-*b*]diazepine-6(11*H*)-ones

**DOI:** 10.1016/j.ejmech.2013.10.052

**Published:** 2013-12

**Authors:** Xianming Deng, Jonathan M. Elkins, Jinwei Zhang, Qingkai Yang, Tatiana Erazo, Nestor Gomez, Hwan Geun Choi, Jinhua Wang, Nicolas Dzamko, Jiing-Dwan Lee, Taebo Sim, NamDoo Kim, Dario R. Alessi, Jose M. Lizcano, Stefan Knapp, Nathanael S. Gray

**Affiliations:** a Department of Cancer Biology, Dana-Farber Cancer Institute, Harvard Medical School, 250 Longwood Ave, SGM 628, Boston, MA 02115, USA; b Department of Biological Chemistry & Molecular Pharmacology, Harvard Medical School, 250 Longwood Ave, SGM 628, Boston, MA 02115, USA; c Structural Genomics Consortium, Nuffield Department of Clinical Medicine and Target Discovery Institute (TDI), University of Oxford, Oxford, UK; d MRC Protein Phosphorylation Unit, College of Life Sciences, University of Dundee, Dow Street, Dundee DD1 5EH, Scotland, UK; e Department of Immunology and Microbial Science, The Scripps Research Institute, 10550 North Torrey Pines Road, La Jolla, CA 92037, USA; f Institut de Neurociències, Departament de Bioquímica i Biologia Molecular, Universitat Autònoma de Barcelona, E-08193 Barcelona, Spain; g Future Convergence Research Division, Korea Institute of Science and Technology, 39-1 Hawologok-Dong, Wolsong-Gil5, Seongbuk-Gu, Seoul, 136-791, South Korea

**Keywords:** ERK5 inhibitor, Kinase selectivity, Benzo[*e*]pyrimido-[5,4-*b*]diazepine-6(11*H*)-one, BMK1, big MAP kinase 1, DIEA, *N*,*N*-diisopropylethylamine, DCAMKL2, doublecortin and CaM kinase-like 2, DMA, *N*,*N*-dimethylacetamide, EGF, epidermal growth factor, ERK5, extracelluar-signal-regulated kinase 5, HCC, hepatocellular carcinoma, LRRK2, leucine rich repeat kinase 2, MAPK, mitogen-activated protein kinase, ERK5, mitogen-activated protein kinase 7, MEK5, MAP kinase kinase 5, Pd_2_(dba)_3_, tris(dibenzylideneacetone)dipalladium-(0), PLK, polo-like kinase, PML, promyelocytic leukemia protein, RSK, ribosomal S6 kinase, SAR, structure–activity relationship, X-phos, 2-dicyclohexylphosphino-2′,4′,6′-triisopropyl-biphenyl

## Abstract

The benzo[*e*]pyrimido-[5,4-*b*]diazepine-6(11*H*)-one core was discovered as a novel ERK5 (also known as MAPK7 and BMK1) inhibitor scaffold, previously. Further structure–activity relationship studies of this scaffold led to the discovery of ERK5-IN-1 (**26**) as the most selective and potent ERK5 inhibitor reported to date. **26** potently inhibits ERK5 biochemically with an IC_50_ of 0.162 ± 0.006 μM and in cells with a cellular EC_50_ for inhibiting epidermal growth factor induced ERK5 autophosphorylation of 0.09 ± 0.03 μM. Furthermore, 26 displays excellent selectivity over other kinases with a KINOME*scan* selectivity score (*S*_10_) of 0.007, and exhibits exceptional bioavailability (F%) of 90% in mice. **26** will serve as a valuable tool compound to investigate the ERK5 signaling pathway and as a starting point for developing an ERK5 directed therapeutic agent.

## Highlights

• Structural determinants of benzo[*e*]pyrimido-[5,4-*b*]diazepine-6(11*H*)-ones for ERK5.
• Highly selective ERK5 inhibitor with good efficacy both *in vitro* and *in vivo*.
• Represents a good template for developing ERK5 directed therapeutic agent.

## Graphical abstract

**Figure:**
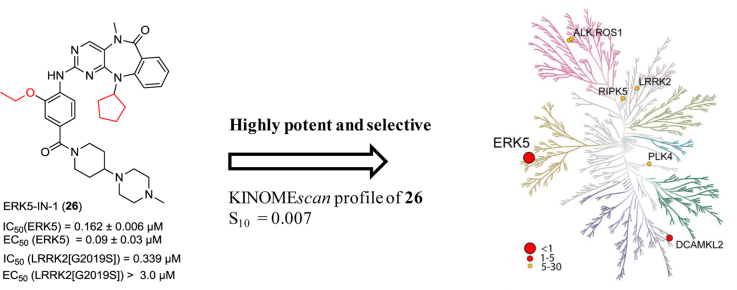

## 1 Introduction

Extracelluar-signal-regulated kinase 5 (ERK5) (also known as MAPK7 and big MAP kinase 1 (BMK1)) is the least well studied member of mitogen-activated protein kinases (MAPKs) family [1,2]. Recent data pointing to a potential role of ERK5 in pathological conditions such as cancer and tumor angiogenesis has greatly increased interest in this signaling pathway [3]. For example, in breast cancer, ERK5 expression is up-regulated by constitutive activation of signal transducer and activator of transcription 3 (STAT3) [4]. In prostate cancer, ERK5 is over-expressed and correlates with the presence of bone metastases and aggressiveness of the disease [5,6]. Additionally, ERK5 is a target for gene amplification at 17p11 in hepatocellular carcinoma (HCC), an amplification detected in approximately 50% of primary HCC tumors [7]. More importantly, reduction of ERK5 expression or signaling significantly inhibited the motility and invasive capability of PC3 cells [8]. And ERK5 protein levels are also regulated by tumor-suppressive microRNAs (miRNAs), including *miR-143* and *miR-145* [9–11]. These observations suggest that small molecule ERK5 inhibitors may serve as potential therapeutic agents for the treatment of ERK5-dependent cancer and other diseases.

**Fig. 1:**
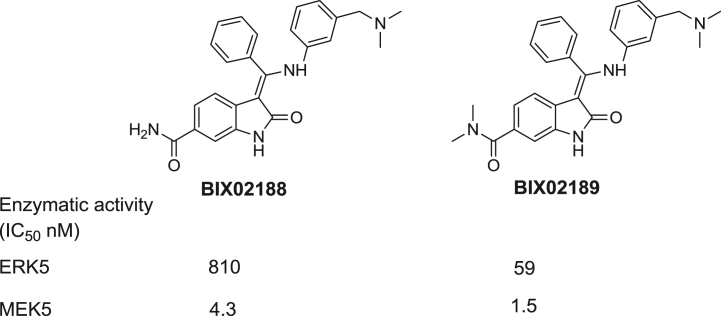
Dual inhibitors of ERK5 and MEK5.

**Fig. 2:**
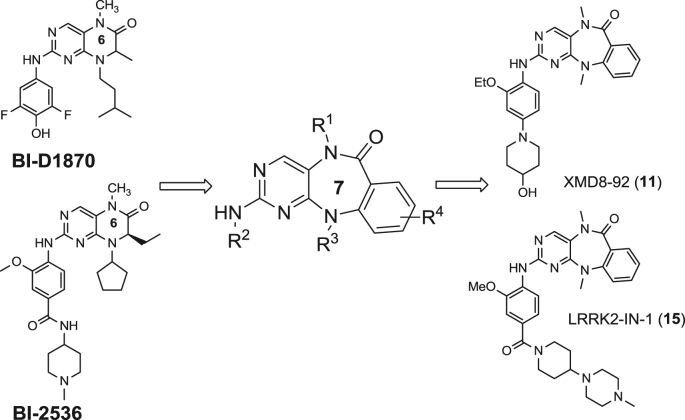
Development of ERK5 inhibitor (XMD8-92) and LRRK2 inhibitor (LRRK2-IN-1) from pyrimido-diazepine.

Despite the substantial progress in elucidating the functions of ERK5, the development of selective ERK5 inhibitors has lagged behind. Since the cloning of ERK5 in 1995 [12,13], there have been only two reported oxindole derived inhibitors which are dual inhibitors of ERK5 and its upstream kinase MEK5: BIX02188 and BIX02189 (Fig. 1) [14]. In an effort to discover small molecules that can selectively inhibit ERK5 kinase activity, we designed and synthesized a collection of approximately fifty analogs derived from the 2-amino pyrido[2,3-d]pyrimidine template. The pyrido[2,3-d]pyrimidine core can be classified as a privileged ATP-site targeting scaffold as is exemplified by compounds such as: BI-2536, a selective polo-like kinase family (PLK1, PLK2 and PLK3) inhibitor [15,16] and BI-D1870, an inhibitor of RSK [17] (Fig. 2). Kinome-wide selectivity profiling of this collection of 2-amino pyrido[2,3-d]pyrimidines using the KINOME*scan* approach [18] resulted in the discovery of benzo[*e*]pyrimido-[5,4-*b*]diazepine-6(11*H*)-ones as inhibitors of ERK5. In our previous study (Fig. 2) [19–21], we reported the identification and preliminary structure–activity relationship (SAR) of benzo[*e*]pyrimido-[5,4-*b*]diazepine-6(11*H*)-one XMD8-92 (**11**) as a selective and moderately potent cellular and *in vivo* ERK5 inhibitor [20]. Using this inhibitor, **11**, we determined that ERK5 inhibits the tumor suppressor activity of cellular promyelocytic leukemia protein (PML) and demonstrated efficacy and tolerability of the inhibitor against two tumor xenografts providing preliminary support for further exploration of ERK5 inhibitors as anti-cancer agents [20]. Later, LRRK2-IN-1 (**15**), a new analog derived from this scaffold, was identified as a leucine rich repeat kinase 2 (LRRK2) inhibitor with potent activity and a good selectivity profile using a “compound centric” kinase profiling strategy [21]. Compound **15** also exhibited a cellular EC_50_ for inhibiting epidermal growth factor (EGF) induced ERK5 autophosphorylation of 0.16 μM. These results demonstrated that the benzo[e]pyrimido-[5,4-*b*]diazepine-6(11*H*)-one scaffold is well suited to developing inhibitors of both ERK5 and LRRK2 and we were curious to explore modifications to the scaffold that could impart selectivity between these two targets and across the kinome in general. In this article, we describe biochemical and cellular analysis of the benzo[*e*]pyrimido-[5,4-*b*]diazepine-6(11*H*)-one scaffold with respect to their activity against ERK5 and LRRK2. This effort culminated in the discovery of ERK5-IN-1 (**26**) as the most potent and selective ERK5 inhibitor reported to date.

## 2 Results and discussion

### 2.1 Chemistry

**Scheme 1:**
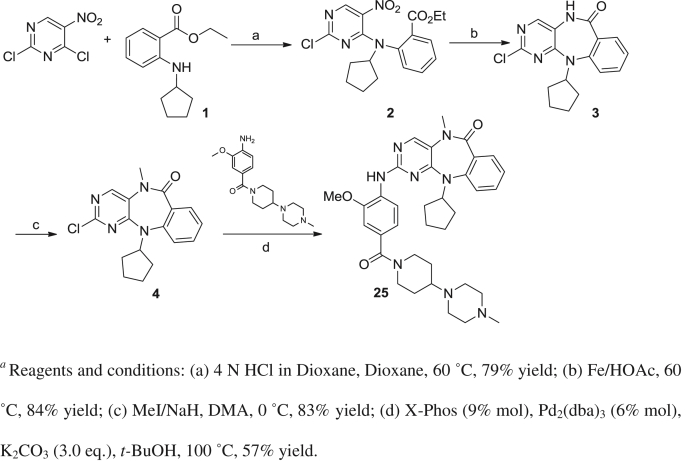
Synthesis of 2-Amino-11-cyclopentyl-5-methyl-5H-benzo[*e*]pyrimido[5,4-*b*][1,4] diazepin-6(11*H*)-one.^a^ ^a^Reagents and conditions: (a) 4 N HCl in Dioxane, Dioxane, 60 °C, 79% yield; (b) Fe/HOAc, 60 °C, 84% yield; (c) MeI/NaH, DMA, 0 °C, 83% yield; (d) X-Phos (9% mol), Pd_2_(dba)_3_ (6% mol), K_2_CO_3_ (3.0 eq.), *t-*BuOH, 100 °C, 57% yield.

An efficient four-step synthetic route was developed to enable the synthesis of benzo[*e*]pyrimido-[5,4-*b*]diazepine-6(11*H*)-ones. Compounds **5**–**18** and **20**–**24** were synthesized as described previously [19]. The synthesis of **25** using a modified synthetic procedure is outlined in Scheme 1. First, 2,4-dichloro-5-nitropyrimidine was reacted with *N*-cyclopentylanthranilic ethyl ester under acidic conditions using 4 N hydrochloric acid in dioxane at 60 °C to give the amination product **2** in good yield. We observed that the yield of the substitution reaction under acidic conditions is higher than that obtained under basic condition which we used previously [19]. The substitution reaction was followed by iron-mediated reduction of **2** and *in situ* cyclization in acetic acid at 60 °C to afford the 7-member lactam intermediate **3** in good yield. Compound **25** was obtained via methylation of the lactam of **3** followed by palladium mediated amination of **4** with (4-amino-3-methoxyphenyl) (4-(4-methylpiperazin-1-yl)piperidin-1-yl)methanone.

### 2.2 SAR of benzo[*e*]pyrimido-[5,4-*b*]diazepine-6(11*H*)-ones for ERK5 and LRRK2

The SAR of benzo[*e*]pyrimido-[5,4-*b*]diazepine-6(11*H*)-ones was explored with respect to ERK5 and LRRK2 using a combination of biochemical and cellular readouts. We used a cellular assay that measures the ability of compounds to inhibit the autophosphorylation of ERK5 in HeLa cells in response to epidermal growth factor (EGF) stimulation. HeLa cells were serum starved overnight followed by treatment with inhibitors for 1 h. Cells were then stimulated with epidermal growth factor (EGF, 20 ng/mL) for 17 min, and ERK5 activation was detected by mobility retardation [22]. Here we also used a newly developed radiochemical assay to measure inhibition of recombinant active ERK5, using ^32^P-ATP and the PIMtide peptide as substrates. Compounds were tested for their ability to inhibit the incorporation of ^32^P into the PIMtide substrate. We assessed inhibition of LRRK2 by measuring the IC_50_ values of the inhibitors against recombinant LRRK2 [G2019S], a naturally occurring activating mutation linked to Parkinsons' disease [23], using the Adapta kinase assay format (Invitrogen) as readouts (Tables 1 and 2) [24]. Our cellular assay for LRRK2 measures the ability of compounds to inhibit LRRK2-dependent phosphorylation of Ser910 and Ser935 in stably transformed HEK293 cells as described previously [21,25].

We first explored the 2-amino moiety of this scaffold by introducing different anilines and alkyl amines. Introduction of 2-methoxy-4-(4-methylpiperazin-1-yl)aniline, 2-methoxy-4-(4-hydroxypiperidin-1-yl)aniline, 2-methoxy-4-morpholinoaniline and 4-sulfonamideaniline resulted in compounds **5**–**8**. These compounds all exhibited potent inhibition of ERK5 with cellular EC_50_ values of 0.19–0.31 μM and enzymatic IC_50_ values of 0.09–0.30 μM and of LRRK2[G2019S] with enzymatic IC_50_ values of 0.007–0.031 μM (Table 1). In contrast introduction of a 2-(1H-imidazol-2-yl)ethanamine or 1-methylpiperidin-4-amine as in compounds **9** and **10** resulted in complete loss of activity against both ERK5 and LRRK2[G2019S]. These results suggested that the phenyl ring of the 2-amino moiety may possess important interactions with both kinases. We investigated the effect of *ortho*-substitution of the aniline by introducing a 2-ethoxy (**11**) and 2-isoproxy (**12**). Compound **11** possessed similar activity against ERK5 comparing to **6** and exhibited slightly decreased activity against LRRK2[G2019S]. Compound **12** which contains the 2-isoproxyl substituent exhibited a dramatic decrease in activity for both kinases. These observations combined with published SAR for a pan Aurora inhibitor from the same scaffold [26] and the ERK5 inhibitor XMD8-92 (**11**) [19] suggest that substitution of the *ortho*-position of the 2-aniline moiety of **5** with larger substituents is slightly better tolerated for ERK5 than for LRRK2. Introduction of various amides at the 4-position of 2-methoxylaniline resulted in compounds **13**–**15**. Compounds **13** and **14** maintained similar activity against ERK5 while **15** exhibited the best activity within this set of compounds. Meanwhile compounds **13**–**15** exhibited IC_50_ values in the single digit nanomolar range against LRRK2[G2019S]. In sum, the amide functional group at the 4-position of the 2-anilino moiety is favorable for both ERK5 and LRRK2.

We next investigated the effects of modification to the N-substituent (**R**^**2**^) of the lactam, to the N-substituent (**R**^**3**^) of the anthranilic acid and to the aryl ring (**R**^**4**^) (Table 2). Compound **16** containing an unsubstituted lactam amide exhibited a 4-fold decrease in activities for both ERK5 and LRRK2[G2019S] kinases, which indicated the methyl substitution of the lactam is preferred. N-substituents (**R**^**3**^) of increasing size from methyl (**5**), ethyl (**17**), isopropyl (**18**), to cyclopentyl (**19**) all maintained similar ERK5 inhibitory activity while inhibition of LRRK2[G2019S] was diminished. This series resulted in the important insight that ERK5 can tolerate larger **R**^**3**^ groups relative to LRRK2[G2019S] thereby providing a method to achieve selectivity for ERK5. For example, compound **19** containing a *N*-cyclopentyl at **R**^**3**^ exhibited a 30-fold decrease in IC_50_ (0.781 μM) for LRRK2[G2019S] relative to the methylated compound **5**. There appears to be limited tolerance for substitution on the aryl ring of anthranilic acid, as the 4-fluoro, 5-chloro, 4-chloro, and 5-methyl analogs (**20**, **21**, **22**, and **23** respectively) all exhibited a dramatic loss in activity for both ERK5 and LRRK2. Compound **24** with the indoline-7-carboxylic linkage (**R**^**6**^) exhibited similar LRRK2 activity compared to LRRK2-IN-1 (**15**), while the ERK5 activity was decreased. However, the use of **24** at a concentration of 1 μM in cellular assays is still likely to inhibit both targets. Compound **25** having the combination of 2-methoxy-4-(4-methylpiperazin-1-yl)piperidin-1-carbonyl-aniline (**R**^**2**^) and *N*-cyclopentyl (**R**^**3**^) exhibited a cellular EC_50_ of 0.08 ± 0.02 μM and an enzymatic IC_50_ of 0.082 ± 0.009 μM against ERK5 and an enzymatic IC_50_ of 0.061 μM for LRRK2[G2012S] which is more than a 10-fold decrease in activity against this target when compared to LRRK2-IN-1 (**15**). Further elaboration to combine the *ortho*-ethoxy substitution and the cyclopentyl at **R**^**3**^ resulted in the synthesis of **26** which possesses a cellular EC_50_ of 0.09 ± 0.03 μM and an enzymatic IC_50_ of 0.162 ± 0.006 μM against ERK5 and enzymatic IC_50_ of 0.339 μM against LRRK2[G2019S]. Compound **26** represents the most potent and selective ERK5 inhibitor that we have been able to discover from this chemical series. The ability of the benzo[*e*]pyrimido-[5,4-*b*]diazepine-6(11*H*)-ones to inhibit ERK5 activity *in vitro* correlated well with their ability to inhibit ERK5 autophosphorylation in cells (see the scatter plot in Supplemental Fig. S1).

The SAR exploration of the benzo[*e*]pyrimido-[5,4-*b*]diazepine-6(11*H*)-one scaffold led to the discovery of the relatively LRRK2 selective inhibitor **24** and ERK5 selective inhibitor **26**. The structural features of *N*-methyl substitution of the lactam (**R**^**2**^), the 2-ethoxy group of the 4-amide substituted aniline, *N*-cyclopentyl substitution (**R**^**3**^) of the anthranilic acid and no substituent (**R**^**4**^ = *H*) on the aryl ring of anthranilic acid were essential to achieve potent cellular inhibitory activity against ERK5 and high specificity.

### 2.3 Cellular LRRK2 inhibitory effect of compound **24** and **26**

**Fig. 3:**
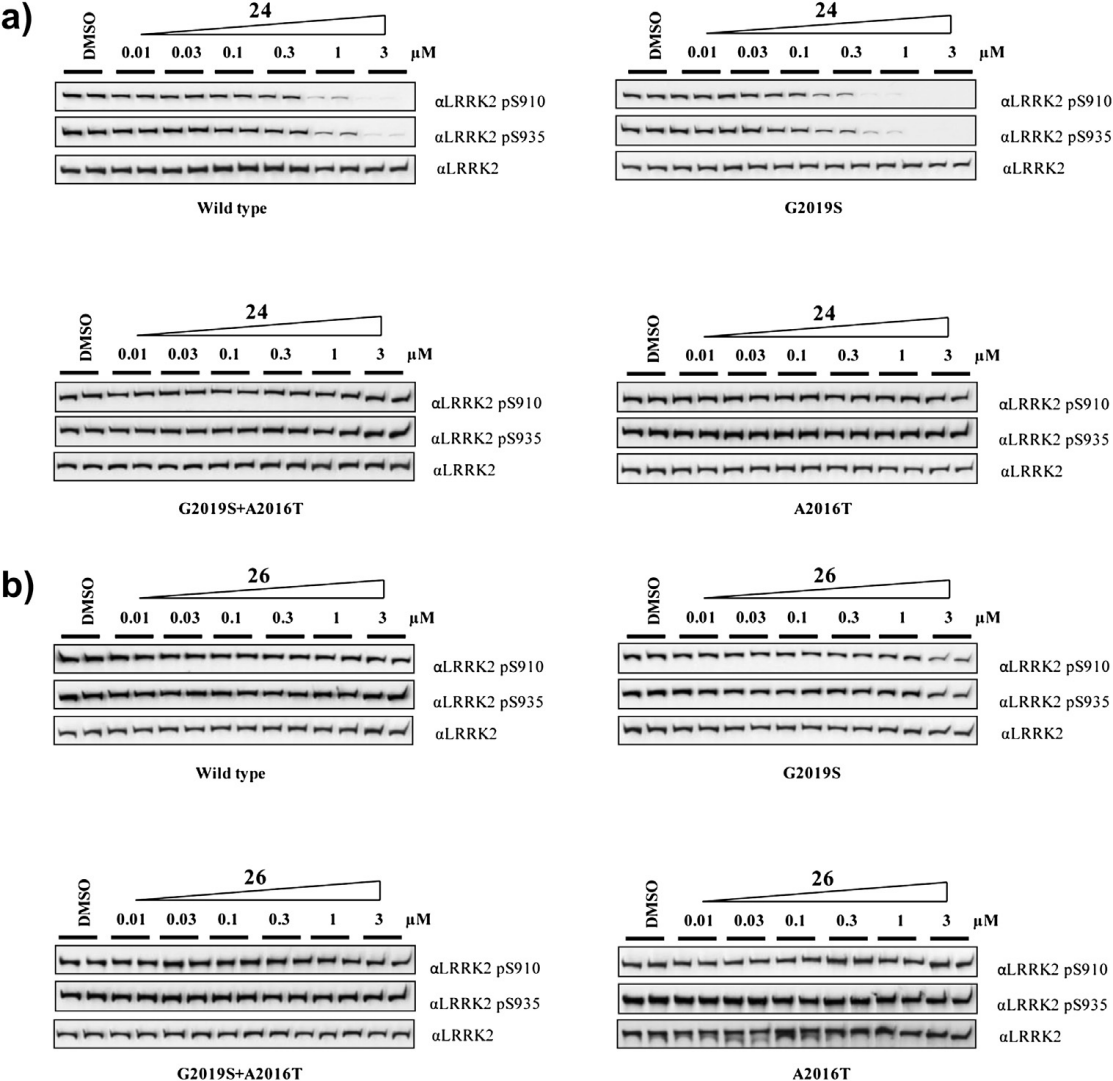
Compound **24** inhibits LRRK2 in cells, but **26** not. a) HEK293 cells stably expressing wild-type GFP-LRRK2, GFP-LRRK2[G2019S], GFP-LRRK2[G2019S + A2016T], and GFP-LRRK2[A2016T] were treated with DMSO or increasing concentrations of compound **24** for 90 min. Cell lysates were subjected to immunoblotting for detection of LRRK2 phosphorylated at Ser910 and Ser935 and for total LRRK2. b) As in a) except **26** was used at the indicated concentration.

We examined the abilities of compounds **24** and **26** to inhibit LRRK2 in a cellular context. As there are no validated direct phosphorylation substrates of LRRK2, we monitored phosphorylation of Ser910 and Ser935, two residues whose phosphorylation is known to be dependent upon LRRK2 kinase activity [25] (Fig. 3). Compound **24** induced a dose-dependent inhibition of Ser910 and Ser935 phosphorylation in both wild-type LRRK2 and LRRK2[G2019S] stably transfected HEK293 cells (Fig. 3a). Significant reduction on the level of phosphorylation of residues Ser910 and Ser935 was observed at 1–3 μM of **24** for wild-type LRRK2 and at slightly lower doses for LRRK2[G2019S] (Fig. 3a), which is comparable to the potency exhibited by LRRK2-IN-1 [21]. Compound **24** had no effect on the phosphorylation of Ser910 and Ser935 at a concentration of up to 3 μM in the drug-resistant LRRK2[G2019S + A2016T] and LRRK2[A2016T] mutants (Fig. 3a), revealing that **24** has the same activity profile as LRRK2-IN-1 [21]. Consistent with the biochemical results, compound **26** did not show any inhibitory effect against LRRK2 at a concentration of up to 3 μM in this cellular context (Fig. 3b).

**Fig. 4:**
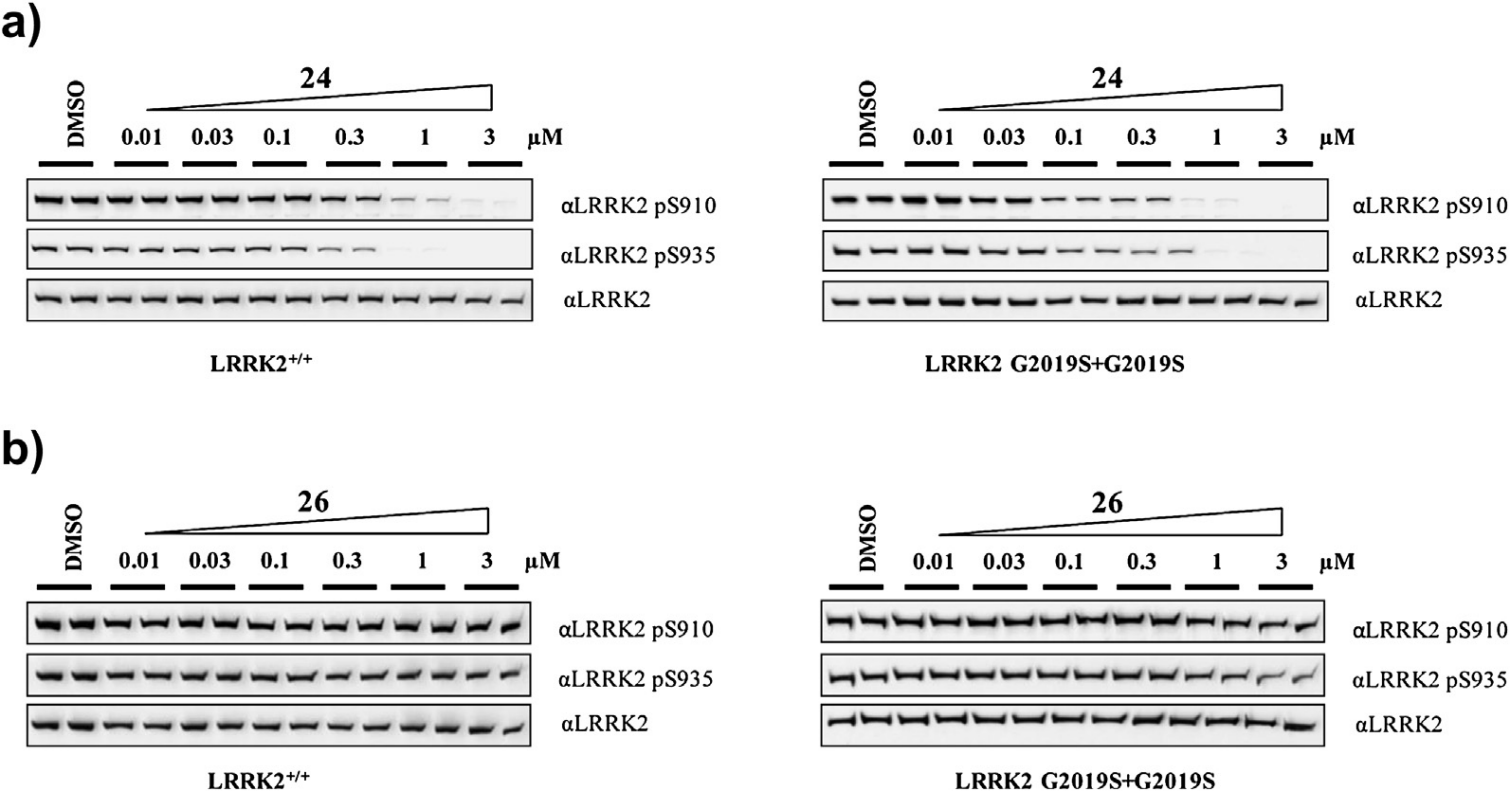
Compound **24** effectively inhibits endogenously expressed LRRK2, but compound **26** not. Endogenous LRRK2 from EBV immortalized human lymphoblastoid cells from a control subject and a Parkinson's disease patient homozygous for the LRRK2[G2019S] mutation. After treatment of the cells with DMSO or the indicated concentration of compound 24 (or 26) for 90 min, cell lysates were subjected to immunoblot analysis with the purified indicated antibody for western analysis. Immunoblots were performed in duplicate, and results were representative of at least two independent experiments.

We next examined the effects of compounds **24** and **26** on endogenously expressed LRRK2 in human lymphoblastoid cells derived from a control and Parkinson's disease patient homozygous for the LRRK2[G2019S] mutation (Fig. 4). We found that increasing doses of **24** led to similar reduction on the levels of phosphorylation of endogenous LRKK2 at Ser910 and Ser935, as was observed in HEK293 cells stably expressing wild-type LRRK2 or LRRK2[G2019S] (compare Figs. 3a to 4a). Moreover, **24** was also more potent against LRRK2[G2019S] mutant than wild type LRRK2, which is consistent with the trend we observed in HEK293 cells. Similarly, compound **26** did not show inhibitory effects on endogenous LRRK2 (compare Figs. 3b to 4b). Taken together, compound **24** is as potent LRRK2 inhibitor as LRRK2-IN-1 and worked both *in vitro* and *in cells* and with improved selectivity towards LRRK2. Compound **26** is an ERK5 specific inhibitor, which has at least 30-fold cellular selectivity for ERK5 relative to LRRK2 and should not inhibit LRRK2 when used at 1 μM concentrations.

The pharmacokinetic properties of **26** were also evaluated following intravenous and oral delivery in mice. This study demonstrated that **26** exhibits favorable pharmacokinetic properties with a T_1/2_ of 8.2 h, AUC of 15745 h*ng/mL and %F of 90 (Table 3).

### 2.4 Kinase selectivity analysis of compounds **24**, **25** and **26**

We assessed the selectivity of members of this scaffold using the KINOME*scan* methodology across a near comprehensive panel of 442 kinases [18,27]. Compounds **24**, **25** and **26** were screened at a concentration of 10 μM which revealed a highly selective profile for this inhibitor class (see data in the Supplementary Material). Compound **26**, which contains an *ortho*-ethoxy aniline demonstrated outstanding selectivity with a KINOME*scan* selectivity score of S_10_ of 0.007 (3/442), and only interactions with ERK5, doublecortin and CaM kinase-like 2 (DCAMKL2) and polo-like kinase 4 (PLK4) were detected. Compound **25** containing a *ortho*-methoxy aniline exhibited a S_10_ of 0.018 (8/442). These results revealed that the ortho-substituent could serve as a selectivity handle. Compared with our previously reported ERK5 inhibitor, XMD8-92 (**11**, S_10_ = 0.012, 5/402) [21], compound **26** represents a further improvement in selectivity. Compound **24** exhibited the same KINOME*scan* selectivity score of S_10_ of 0.036 (16/442) as that of our previous LRRK2 inhibitor (LRRK2-IN-1) [21], while being more selective for LRRK2 relative to ERK5. Compounds **25** and **26** were also profiled against selected panels of kinases in HeLa and PC3 cell lysates using a chemical proteomics approach, KiNativ [28]. These results revealed that only ERK5 was inhibited with higher than 90% target occupancy at a concentration of 10 μM for both **25** and **26**, which further confirmed their highly selective profiles (Please see Supplementary profiling data for details).

**Fig. 5:**
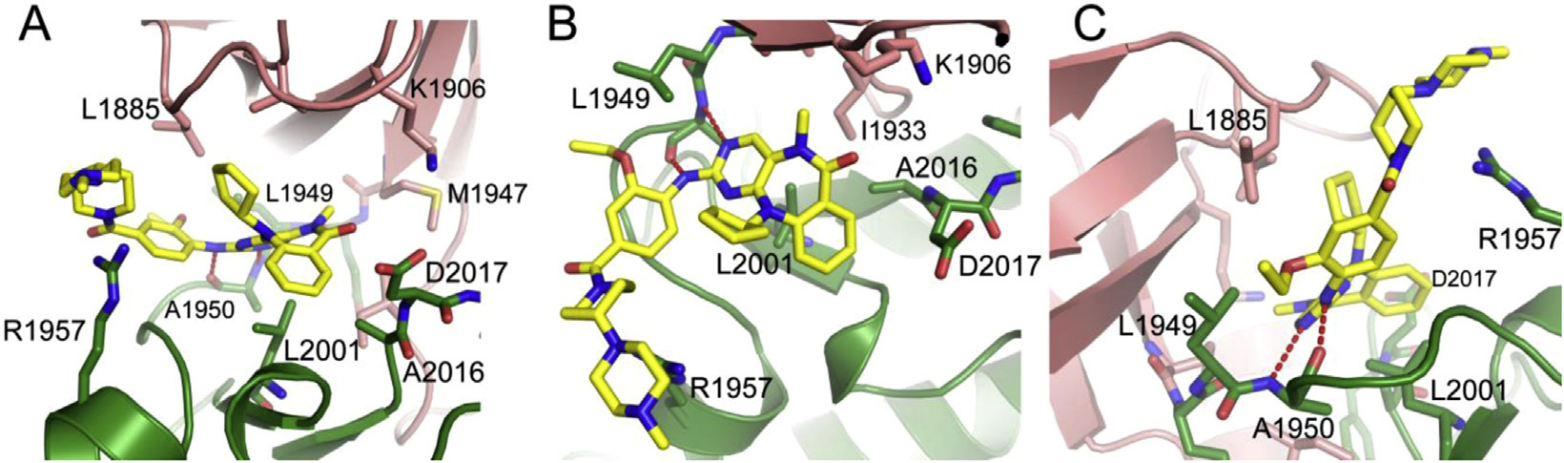
Docking model of **26** bound to LRRK2 from three different viewing angles. The N-terminal lobe of the LRRK2 model is shown in pink, and the C-terminal lobe in green. **26** is shown in yellow. (For interpretation of the references to colour in this figure legend, the reader is referred to the web version of this article.)

To better understand the SAR for LRRK2, we performed a molecular modeling study using Glide [29]based upon the recently reported crystal structure of Roco kinase (PDB accession code: 4F1T [30]) (Fig. 5). This model allows explanation of some of the SAR that we observed. Overall **26** is predicted to bind to LRRK2 in a manner analogous to what has been observed for a structural analog, Mps1-IN-2, bound to TTK [31]: The tricyclic core of the compound curves around Leu2001 in the base of the ATP binding site, forming two hydrogen bonds with the hinge region at Ala1950, while the piperidin-piperazine points towards the solvent exposed region (Fig. 5A). The cyclopentyl group points towards the glycine rich loop, against Leu1885, and would appear to force the tricyclic ring towards the base of the ATP binding site, making contact with Ala2016. These observations are consistent with the SAR results of decreased LRRK2 affinity with increasing size up to cyclopentyl (**5**, **17**, **18**, **19**) (Fig. 5B) of the anthranilic acid N-substituent. The same contacts with Ala2016 and nearby residues would explain why substitution of the anthranilic acid (**20**, **21**, **22**, and **23**) results in weaker binding. The unfavorable interaction between the *ortho*-substituent of the aniline with Leu1949 increases as the substituent goes from methoxy to ethoxy to isopropoxyl (**6**, **11**, and **12**) (Fig. 5C), which resulted in decreased affinity for LRRK2. These key active site residues of LRRK2 are either conserved in ERK5 (Leu1949, Leu2001) or conservatively substituted (Leu1885, Ala2016, Met1947). Our recently determined co-crystal structure of ERK5 with **25** also confirms both the binding model for **26** with ERK5 and the observed SAR trends [32].

## 3 Conclusions

The new chemo-type of benzo[*e*]pyrimido-[5,4-*b*]diazepine-6(11*H*)-one represents a privileged scaffold for developing ERK5 and LRRK2 kinase inhibitors. A comprehensive SAR exploration led to the identification of the key structural features required to separate the SAR of this scaffold between ERK5 and LRRK2. Compound **24** is as potent a LRRK2 inhibitor as LRRK2-IN-1 with improved selectivity for LRRK2 relative to ERK5 that also possesses activity in both *in vitro* and cellular assays. Compound **26** represents the most selective and potent ERK5 inhibitor we have developed so far. Given the outstanding specificity, excellent cellular efficacy and favorable pharmacokinetic properties, **26** should serve as a versatile tool to further probe ERK5 biology. The benzo[*e*]pyrimido-[5,4-*b*]diaze pine-6(11*H*)-ones scaffold represents a ‘priviledged’ template for kinase inhibition due to its ability to be engineered to possess excellent kinase selectivity, favorable pharmacokinetic parameters [20,21], and efficacy in xenograft tumor models [20].

## 4 Experimental protocols

### 4.1 Chemistry general procedures

Unless otherwise noted, reagents and solvents were obtained from commercial suppliers and were used without further purification. ^1^H NMR spectra were recorded on 600 MHz (Varian AS600), and chemical shifts are reported in parts per million (ppm, *δ*) downfield from tetramethylsilane (TMS). Coupling constants (*J*) are reported in Hz. Spin multiplicities are described as s (singlet), brs (broad singlet), t (triplet), q (quartet), and m (multiplet). Mass spectra were obtained on a Waters Micromass ZQ instrument. Preparative HPLC was performed on a Waters Symmetry C18 column (19 × 50 mm, 5 μM) using a gradient of 5–95% acetonitrile in water containing 0.05% trifluoroacetic acid (TFA) over 8 min (10 min run time) at a flow rate of 30 mL/min. Purities of assayed compounds were in all cases greater than 95%, as determined by reverse-phase HPLC analysis.

### 4.2 Synthesis of 11-cyclopentyl-2-((2-methoxy-4-(4-(4-methylpiperazin-1-yl)piperidine-1-carbonyl)phenyl)amino)-5-methyl-5*H*-benzo[*e*]pyrimido[5,4-*b*][1,4]diazepin-6(11*H*)-one (**25**)

A mixture of ethyl 2-(cyclopentylamino)benzoate (1.40 g, 6.0 mmol), 4 N HCl in dioxane solution (2.25 mL, 9.0 mmol) and 2,4-dichloro-5-nitropyrimidine (1.74 g, 9.0 mmol) in dioxane (40 mL) was heated at 60 °C for 90 h. After the reaction was complete as monitored by thin layer chromatography (TLC), the reaction solution was concentrated and the residue was purified by silica-gel column chromatography with ethyl acetate and hexane (1/20, v/v) to give the amination product **2** (1.84 g, 79%). MS (ESI) *m*/*z* 391 (M + H)^+^.

A mixture of compound **2** (1.79 g, 4.59 mmol) and iron power (2.57 g, 45.9 mmol) in acetic acid (80 mL) was heated at 60 °C for 9 h. After the reaction was complete as monitored by reverse phase analytical liquid-chromatography electrospray mass spectrometry (LC-MS), the solvent was removed *in vacuo*. The resulting residue was poured into ice-water which resulted in a solid precipitate that was collected by filtration, washed with water and air dried to give the intermediate **3** (1.21 g, 84%). ^1^H NMR (600 MHz, DMSO-*d*_*6*_) *δ* 10.48 (s, 1H), 8.18 (s, 1H), 7.57 (d, *J* = 7.2 Hz, 1H), 7.50 (s, 1H), 7.30 (d, *J* = 7.2 Hz, 1H), 7.17 (s, 1H), 4.61 (brs, 1H), 2.30–1.90 (m, 2H), 1.70–1.40 (m, 4H), 1.38–1.20 (m, 2H). MS (ESI) *m*/*z* 315 (M + H)^+^.

To a stirred suspension of compound **3** (314 mg, 1.0 mmol) and MeI (0.13 mL, 2.0 mmol) in dimethyl acetamide (DMA, 10.0 mL) was added NaH (80 mg, 60% suspension in mineral oil) at −10 °C and the reaction was gradually warmed to 0 °C. After the reaction was complete as monitored by LC–MS, the solution was poured into ice-water which resulted in a solid precipitate. The precipitate was collected by filtration, washed with water and air dried to give the intermediate **4** (273 mg, 83%). ^1^H NMR (600 MHz, DMSO-*d*_*6*_) *δ* 8.64 (s, 1H), 7.58 (dd, *J* = 1.8, 7.8 Hz, 1H), 7.49 (t, *J* = 7.8 Hz, 1H), 7.33 (d, *J* = 8.4 Hz, 1H), 7.19 (t, *J* = 7.2 Hz, 1H), 4.68–4.64 (m, 1H), 3.44 (s, 3H), 2.28–2.20 (m, 1H), 2.10–2.02 (m, 1H), 1.64–1.54 (m, 4H), 1.50–1.34 (m, 2H). MS (ESI) *m*/*z* 329 (M + H)^+^.

A mixture of **4** (33 mg, 0.1 mmol), (4-amino-3-methoxyphenyl)(4-(4-methylpiperazin-1-yl)piperidin-1-yl)methanone (33 mg, 0.1 mmol), X-Phos (4.3 mg), Pd_2_(dba)_3_ (5.5 mg) and K_2_CO_3_ (41.5 mg, 0.3 mmol) in 1.2 mL of *t*-BuOH was purged with Argon. The resulting mixture in a seal tube was heated at 100 °C until the reaction was complete as monitored by LC-MS. Then the reaction was filtered through Celite and eluted with dichloromethane. The dichloromethane was removed *in vacuo* and the resulting crude product was purified by reverse-phase prep-HPLC using a water (0.05% TFA)/acetonitrile (0.05% TFA) gradient to afford the title compound **25** (35.3 mg, 57%). ^1^H NMR (400 MHz, DMSO-*d*_*6*_) *δ* 8.44 (s, 1H), 8.20 (d, *J* = 8.0 Hz, 1H), 8.14 (s, 1H), 7.56 (d, *J* = 7.8 Hz, 1H), 7.45 (t, *J* = 7.7 Hz, 1H), 7.28 (d, *J* = 8.2 Hz, 1H), 7.15 (t, *J* = 7.5 Hz, 1H), 7.03 (s, 1H), 7.00 (d, *J* = 8.2 Hz, 1H), 4.69–4.64 (m, 1H), 3.86 (s, 3H), 3.42 (s, 3H), 3.32 (d, *J* = 0.7 Hz, 7H), 2.9–3.1 (m, 6H), 2.75 (s, 3H), 2.28 (m, 1H), 2.07 (m, 1H), 1.75 (br, 2H), 1.60–1.34 (m, 8H); ^13^C NMR (100 MHz, DMSO-d6) *δ* 169.2, 167.7, 164.3, 158.2, 156.2, 152.7, 149.5, 148.7, 132.3, 131.3, 130.3, 128.7, 124.7, 123.7, 121.6, 119.7, 119.2, 110.2, 61.0, 57.1, 56.5, 53.4, 46.3, 42.6, 37.2, 33.1, 32.2, 24.6, 24.5. MS (ESI) *m*/*z* 625 (M + H)^+^, HRMS (ESI) *m*/*z* calc. 625.3615, measured 625.3633 (M + H)^+^.

### 4.3 ERK5 autophosphorylation assay [22]

HeLa cells were serum starved overnight followed by treatment with inhibitors for 1 h. Cells were then stimulated with EGF (20 ng/mL) for 17 min and harvested in RIPA buffer (1× PBS, 1% NP40, 0.5% sodium deoxycholate, 0.1% SDS, 0.1 mg/ml PMSF and 1 mM sodium orthovanadate). Proteins from total cell lysates were resolved by 6% sodium dodecyl sulfate (SDS)-poly-acrylamide gel electrophoresis (PAGE), transferred to nitrocellulose membrane, blocked in 5% nonfat milk, and blotted with anti-ERK5 antibody.

### 4.4 Baculovirus expression of active ERK5 and purification

pFastBAC vector encoding N-terminal hexahistidine-tagged human ERK5 and HA-tagged human MEK5-DD (constitutively active) were used to generate recombinant baculovirus using the Bac-to-Bac system (Invitrogen). *Spodoptera frugiperda* 21 cells (1.5 × 10^6^/ml) were infected at a multiplicity of infection of 6 with a mix of both baculovirus and harvested 72 h post-infection. Pelleted cells were lysed in ice-cold lysis buffer (50 mM Tris/HCl, pH 7.5, 1 mM EGTA, 1 mM EDTA, 1 mM sodium orthovanadate, 10 mM sodium β-glycerophosphate, 50 mM NaF, 5 mM sodium pyrophosphate, 0.27 M sucrose, 1 mM benzamidine, 2 mM phenylmethanesulphonylfluoride (PMSF) and 1% Triton X-100), lysed in one round of freeze/thawing, sonicated (4 × 20 s) and centrifuged at 25,000 g for 30 min. His-tagged ERK5 was purified as described for His-tagged BRSK1[33], using 5 mL Ni-NTA-agarose resin (Qiagen) followed by gel filtration chromatography on Superdex 200HR column on an AKTA system (GE Healthcare). Active ERK5 was purified with yields of ∼5 mg/L of infected cells, and was greater than 90% homogeneous as judged by densitometric scanning of Coomassie Blue-stained SDS/PAGE gels.

### 4.5 ERK5 kinase activity *in vitro* assay

Kinase activity was determined in an assay volume of 40 μL in kinase buffer (50 mM Tris–HCl, pH 7.5, 0.1 mM EGTA, 1 mM 2-mercaptoethanol) containing 200 ng of pure active ERK5 and the indicated amount of inhibitor. Reaction started by adding 10 mM magnesium acetate, and 50 μM [γ-^32^P]-ATP (500 cpm/pmol) and 250 μM PIMtide (ARKKRRHPSGPPTA) as substrates. Assays were carried out for 20 min at 30 °C, terminated by applying the reaction mixture onto p81 paper and the incorporated radioactivity measured as described previously [34].

### 4.6 Adaptor kinase assay of LRRK2 [G2019S]

*In vitro* kinase assays were conducted at Invitrogen (Madison, WI) using the SelectScreen Kinase Profiling Service.

### 4.7 LRRK2 cellular assay

Reagents and general methods. Tissue-culture reagents were from Life Technologies. Protein G Sepharose was from Amersham. DNA constructs used for transfection were purified from *Escherichia coli* DH5α using Qiagen or Invitrogen plasmid Maxi kits according to the manufacturer's protocol. All DNA constructs were verified by DNA sequencing, which was performed by The Sequencing Service, School of Life Sciences, University of Dundee, Scotland, U.K., using DYEnamic ET terminator chemistry (Amersham Biosciences) on Applied Biosystems automated DNA sequencers.

Cell culture, treatments and cell lysis. HEK293 was cultured in DMEM (Dulbecco's Modified Eagle's medium) supplemented with 10% FBS (fetal bovine serum), 2 mM glutamine and 1× penicillin/streptomycin solution. Lymphoblastoid cell lines were generated by EBV (Epstein–Barr virus) transformation of B lymphocytes using standard methods (European Collection of Cell Cultures). Cell-line ANK is derived from a 47-year-old individual homozygous for the LRRK2[G2019S] mutation who presented with Parkinson's disease. Cell-line AHE is derived from a 31-year-old individual, lacking mutation at the LRRK2 Gly^2019^ residue, and presented with no disease. Human lymphoblastoid cells were maintained in RPMI 1640 with 10% FBS, 2 mM glutamine, 1× penicillin/streptomycin solution and were maintained at cell density of 0.3 × 10^6^–2 × 10^6^ cells per mL. Epstein–Barr virus immortalized primary human lymphoblastoid cells from one control subject and one Parkinson's disease patient homozygous for the LRRK2 [G2019S] mutation were kindly provided by Alastair Reith (GSK) and have been described previously.^25^For inhibitor experiments, compounds were dissolved in DMSO and utilized at the indicated concentrations. The concentration of DMSO in the culture media did not exceed 1%. Following treatment, cells were washed once with phosphate buffered saline (PBS) buffer and lysed with lysis buffer (50 mM Tris/HCl, pH 7.5, 1 mM EGTA, 1 mM EDTA, 1 mM sodium orthovanadate, 10 mM sodium β-glycerophosphate, 50 mM NaF, 5 mM sodium pyrophosphate, 0.27 M sucrose, 1 mM benzamidine, 2 mM phenylmethanesulphonylfluoride (PMSF) and 1% Triton X-100). When not used immediately, all lysate supernatants were snap-frozen in liquid nitrogen and stored at −80 °C until use. Protein concentrations were determined following centrifugation of the lysate at 16,000× *g* at 4 °C for 20 min using the Bradford method with BSA as the standard. Transient transfection of HEK 293 cells was performed using the polyethyleneimine (PEI) method [35].

### 4.8 Immunoblot procedures

Cell lysates from human lymphoblastoid cells and GFP-LRRK2 expressing stable cell lines were eluted in 65 μl 2× LDS sample buffer (Invitrogen) with final concentration of 1 μg/μl. Following heating at 70 °C for 10 min, 15 μl aliquots were resolved on 8% SDS polyacrylamide gels and transferred to nitrocellulose membranes for detection of LRRK2 phosphorylated at Ser910, LRRK2 phosphorylated at Ser935 and total LRRK2, using purified rabbit monoclonal antibodies (LRRK2 phospho-serine 910 clone, LRRK2 phospho-serine 935 clone and LRRK2 100-500 clone) in PBS with 0.1% sodium azide (Epitomics). Immunoblot films were scanned on an Epson 4990 scanner, and images were managed with Adobe Photoshop.

### 4.9 Molecular docking study

A molecular docking study to elucidate the interaction between the inhibitors with the LRRK2 kinase domain was performed. First, we constructed the homology model structure of the LRRK2 kinase domain. We used a crystal structure of Roco kinase (PDB accession code: 4F1T). Sequence alignment of LRRK2 and template proteins was generated using the Discovery Studio 3.5 package (http://www.accelrys.com). A 3D model structure of LRRK2 was built by using the Modeller in Discovery Studio 3.5 package and was further refined by using the CHARMM force field. Second, compounds **25** and **26** were built using Maestro build panel and minimized using the Impact module of Maestro in the Schrödinger suite program. The LRRK2 structure was minimized using the Protein Preparation Wizard by applying an OPLS force field. For the grid generation, the binding site was defined as the centroid of the ATP binding site. Ligand docking into the active site of LRRK2 was carried out using the Schrödinger docking program, Glide. The best-docked poses were selected as the lowest Glide score. The molecular graphics for the inhibitor binding pocket and refined docking models were generated using PyMol package (http://www.pymol.org).

## Figures and Tables

**Table 1:**
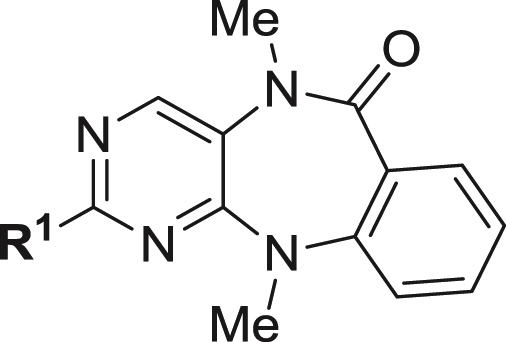
SAR of 2-amino moiety for ERK5 and LRRK2

Compound ID	R^1^	Cellular EC_50_ (ERK5, μM)^a^	Enzymatic IC_50_ (ERK5, μM)^b^	Enzymatic IC_50_ (LRRK2[G2019S], μM)^c^
**5**		0.19 ± 0.04	0.087 ± 0.007	0.026
**6**		0.24 ± 0.04	0.297 ± 0.014	0.017
**7**		0.26 ± 0.03	0.233 ± 0.019	0.031
**8**		0.31 ± 0.06	0.171 ± 0.016	0.07
**9**		4.68 ± 0.52	1.724 ± 0.221	2.37
**10**		4.11 ± 0.59	1.253 ± 0.151	>10
**11**		0.24 ± 0.04	0.364 ± 0.058	0.059
**12**		>0.5	1.530 ± 0.172	0.256
**13**		0.24 ± 0.03	0.140 ± 0.009	0.005
**14**		0.32 ± 0.05	0.338 ± 0.018	0.008
**15**		0.16 ± 0.04	0.114 ± 0.011	0.004

a The required concentration for inhibiting 50% of EGF-stimulated autophosphorylation of ERK5 in HeLa cells.

b The required concentration for inhibiting 50% of enzymatic activity of ERK5 using an *in vitro* assay.

c The required concentration for inhibiting 50% of enzymatic activity of LRRK2[G2019S] using an *in vitro* assay.

**Table 2:**
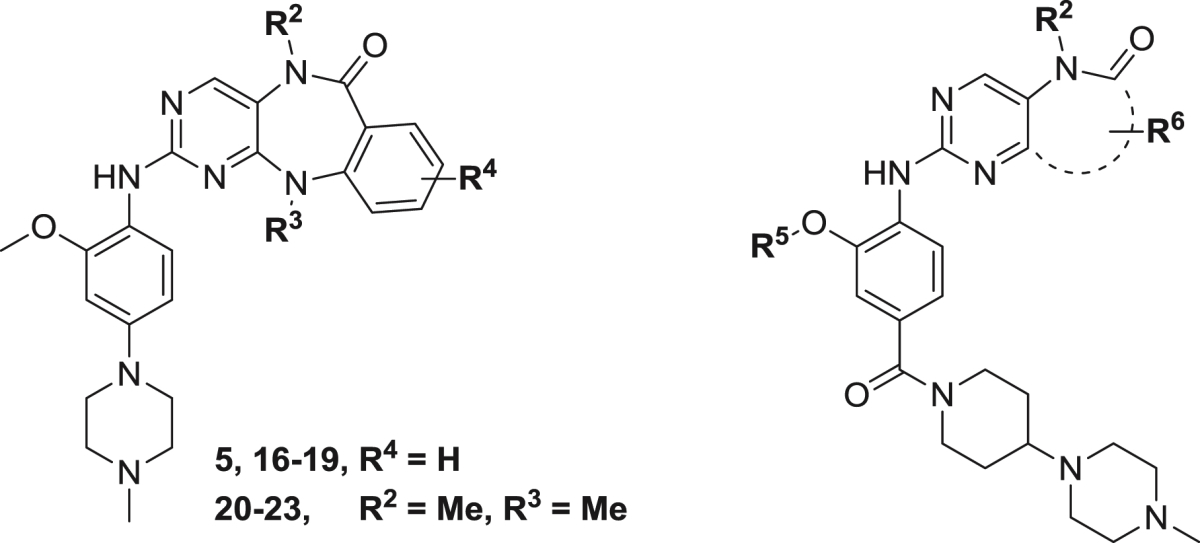
SAR of substituents of anthranilic acid moiety for ERK5 and LRRK2.

Compound ID	**R**	Cellular EC_50_ (ERK5, μM)^a^	Enzymatic IC_50_ (ERK5, μM)^b^	Enzymatic IC_50_ (LRRK2[G2019S], μM)^c^
**5**	R^2^, R^3^ = Me, Me	0.19 ± 0.04	0.087 ± 0.007	0.026
**16**	H, Me	>1.0	1.450 ± 0.210	0.091
**17**	Me, Et	0.20 ± 0.04	0.066 ± 0.007	0.052
**18**	Me, *i*-Pr	0.23 ± 0.03	0.098 ± 0.012	0.36
**19**	Me, cyclopentyl	0.20 ± 0.04	0.146 ± 0.018	0.781
**20**	*R*^4^ = 4-F	1.32 ± 0.22	0.873 ± 0.060	0.439
**21**	5-Cl	1.24 ± 0.24	1.130 ± 0.160	1.07
**22**	4-Cl	11.38 ± 2.04	5.090 ± 0.052	>10
**23**	5-Me	3.10 ± 0.62	2.460 ± 0.260	0.493
**24**		0.26 ± 0.04	0.199 ± 0.012	0.005
**25**		0.08 ± 0.02	0.082 ± 0.009	0.061
**26**		0.09 ± 0.03	0.162 ± 0.006	0.339

a The required concentration for inhibiting 50% of EGF-stimulated autophosphorylation of ERK5 in HeLa cells.

b The required concentration for inhibiting 50% of enzymatic activity of ERK5 using an *in vitro* assay.

c The required concentration for inhibiting 50% of enzymatic activity of LRRK2[G2019S] using an *in vitro* assay.

**Table 3:** Pharmacokinetic parameters of **26**.^a^

Compound	Route	Dose (mg/kg)	*T*_max_ (*H*)	*C*_max_ (ng/mL)	AUC_0−∞_ (h ng/mL)	*T*_1/2_ (hr)	CL (mL/min/Kg)	*V*_ss_ (L/Kg)	*F* (%)
**26**	IV	1	–	439	1743.83	8.2	8.64	4.67	–
PO	10	4.0	1142.77	15745.48	0	–	–	90

a IV = intravenous injection, PO = oral delivery, *T*_max_ = time of maximum plasma concentration, *C*_max_ = maximum plasma concentration, AUC = area under the curve (measure of exposure), *T*_1/2_ = half life, CL = plasma clearance, V_ss_ = volume of distribution, *F* = oral bioavailability.

## Appendix A Supplementary data

Characterization data for compounds **9**, **10**, **12**, **24**, and **26**; KINOME*scan* profiling data for **15**, **24**, **25**, and **26**; KiNativ profiling data for **25** and **26**. This material is available free of charge via the internet at http://www.sciencedirect.com.

### Mol Files

The following ZIP file contains the MOL files of the most important compounds referred to in this article.

MOL file ZIP file containing the MOL files of the most important compounds in this article.
