# Supplementary material for: Structural determinants for ERK5 (MAPK7) and leucine rich repeat kinase 2 activities of benzo[e]pyrimido-[5,4-b]diazepine-6(11H)-ones
Source: Eur J Med Chem. 2013 Dec;70:758–67. doi: 10.1016/j.ejmech.2013.10.052 (PMC3914206; doi:10.1016/j.ejmech.2013.10.052)

Supporting Information

Structural Determinants for ERK5 (MAPK7) and Leucine Rich Repeat Kinase 2 Activities of benzo[e]pyrimido-[5,4-b]diazepine-6(11H)-ones.

Xianming Deng[1], Jonathan M. Elkins[2], Jinwei Zhang[3], Qingkai Yang[4], Tatiana Erazo[5], Nestor Gomez[5], Hwan Geun Choi[1], Jinhua Wang[1], Nicolas Dzamko[3], Jiing-Dwan Lee[4], Taebo Sim[6], NamDoo Kim[6], Dario R. Alessi[3], Jose M Lizcano[5], Stefan Knapp[2] and Nathanael S. Gray[1*]

[1.] Department of Cancer Biology, Dana-Farber Cancer Institute, and Department of Biological Chemistry & Molecular Pharmacology, Harvard Medical School, 250 Longwood Ave, SGM 628, Boston, MA 02115, USA. [2.] Structural Genomics Consortium, Nuffield Department of Clinical Medicine and Target Discovery Institute (TDI), University of Oxford, Oxford, UK. [3] MRC Protein Phosphorylation Unit, College of Life Sciences, University of Dundee, Dow Street, Dundee DD1 5EH, Scotland. [4.] Department of Immunology and Microbial Science, The Scripps Research Institute, 10550 North Torrey Pines Road, La Jolla, CA 92037, USA. [5] Institut de Neurociències, Departament de Bioquímica i Biologia Molecular, Universitat Autònoma de Barcelona, E-08193 Barcelona, Spain. [6] Future Convergence Research Division, Korea institute of Science and Technology, 39-1 Hawologok-Dong, Wolsong-Gil5, Seongbuk-Gu, Seoul, 136-791, Korea.

** To whom correspondence should be addressed. For N.S.G.: phone, 1-617-582-8590; fax, 1-617-582-8615; E-mail, Nathanael_Gray@dfci.harvard.edu .*

Contents

1. Spectral data of compounds **9**, **10**, **12**, **24** and **26**……………………………..………S2
2. Scatter plot of the cellular results and the enzymatic results against MAPK7..................S5
3. Representative ^{1}H NMR and ^{13}C NMR spectra..S7

1. Spectral data of compounds 9, 10, 12 and 24.

Compounds **9**, **10**, **12** and **24** were synthesized by the previous procedures[1].

2-((2-(1H-imidazol-5-yl)ethyl)amino)-5,11-dimethyl-5H-benzo[e]pyrimido[5,4-b][1,4]-diazepin-6(11H)-one (9)

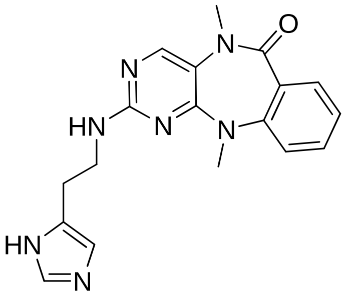

^{1}H NMR (600 MHz, CD_3OD) δ 8.07 (s, 1H), 7.70 (dd, *J* = 1.8, 7.8 Hz, 1H), 7.65 (s, 1H), 7.46 (dt, *J* = 1.8, 7.8 Hz, 1H), 7.16 (d, *J* = 8.4 Hz, 1H), 7.13 (t, *J* = 7.8 Hz, 1H), 6.86 (s, 1H), 3.61 (t, *J* = 7.2 Hz, 2H), 3.41 (s, 3H), 3.31 (s, 3H), 2.88 (t, *J* = 7.2 Hz, 2H).

^{13}C NMR (100 MHz, DMSO-d6) δ 167.8, 159.3, 152.8, 149.9, 135.0, 132.7, 132.0, 116.6, 113.9, 108.0, 41.5, 38.0, 35.5, 26.6. MS (ESI) *m/z* 350 $(M+H)^+$, HRMS (ESI) *m/z* calc. 350.1729, measured 350.1721 $(M+H)^+$.

5,11-dimethyl-2-((1-methylpiperidin-4-yl)amino)-5H-benzo[e]pyrimido[5,4-b][1,4]-diazepin-6(11H)-one (10)

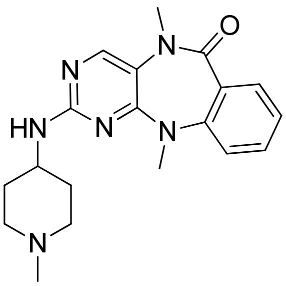

^{1}H NMR (600 MHz, CD_3OD) δ 8.14 (s, 1H), 7.78 (d, *J* = 7.8 Hz, 1H), 7.56 (t, *J* = 7.8 Hz, 1H), 7.28-7.24 (m, 2H), 4.16 (brs, 1H), 3.62 (d, *J* = 12.0 Hz, 2H), 3.47 (s, 3H), 3.44 (s, 3H), 3.20 (t, *J* = 12.6 Hz, 2H), 2.91 (s, 3H), 2.34 (d, *J* = 13.2 Hz, 2H), 1.87 (q, *J* = 13.2 Hz, 2H).

^{13}C NMR (100 MHz, DMSO-d6) δ 167.5, 158.8, 158.4, 158.1, 152.4, 149.7, 132.8, 132.1, 126.7, 123.8, 118.0, 53.3, 43.0, 40.9, 37.9, 35.7, 29.4. MS (ESI) *m/z* 353 $(M+H)^+$. HRMS (ESI) *m/z* calc. 353.2090, measured 353.2102 $(M+H)^+$.

2-((4-(4-hydroxypiperidin-1-yl)-2-isopropoxyphenyl)amino)-5,11-dimethyl-5H-benzo[e]pyrimido[5,4-b][1,4]diazepin-6(11H)-one (12)

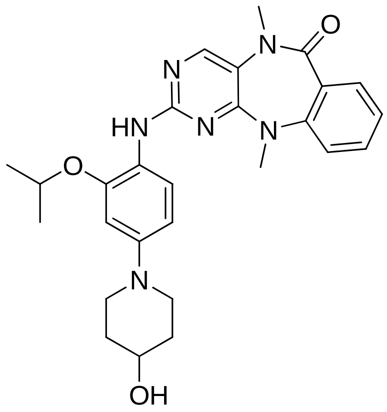

^{1}H NMR (600 MHz, CD_3OD) δ 8.17 (s, 1H), 8.12 (d, *J* = 9.0 Hz, 1H), 7.71 (dd, *J* = 1.2, 7.8 Hz, 1H), 7.45 (dt, *J* = 1.8, 7.8 Hz, 1H), 7.17 (d, *J* = 8.4 Hz, 1H), 7.13 (t, *J* = 7.8 Hz, 1H), 6.67 (d, *J* = 2.4 Hz, 1H), 6.60 (dd, *J* = 3.0, 9.0 Hz, 1H), 4.64-4.60 (m, 1H), 3.74-3.70 (m, 1H), 3.48-3.44 (m, 2H), 3.43 (s, 3H), 3.36 (s, 3H), 2.84-2.80 (m, 2H), 1.97-1.95 (m, 2H), 1.69-1.63 (m, 2H), 1.33 (d, *J* = 6.6 Hz, 6H).

^{13}C NMR (100 MHz, DMSO-d6) δ 167.5, 163.5, 156.6, 152.7, 149.8, 149.5, 148.1, 132.8, 132.1, 126.7, 123.8, 121.9, 121.6, 120.4, 118.2, 108.2, 103.6, 71.2, 66.5, 47.7, 39.9, 36.0, 34.4, 22.3. MS (ESI) *m/z* 489 $(M+H)^+$. HRMS (ESI) *m/z* calc. 489.2614, measured 489.2614 $(M+H)^+$.

2-((2-methoxy-4-(4-(4-methylpiperazin-1-yl)piperidine-1-carbonyl)phenyl)amino)-5-methyl-10,11-dihydropyrimido[4',5':2,3][1,4]diazepino[6,7,1-hi]indol-6(5H)-one (24)

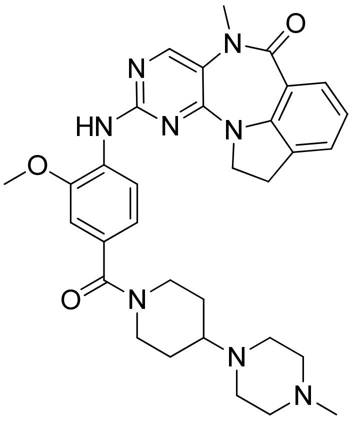

^{1}H NMR (600 MHz, CD_3OD) δ 8.11 (d, *J* = 8.4 Hz, 1H), 7.99 (s, 1H), 7.88 (d, *J* = 7.2 Hz, 1H), 7.44 (dd, *J* = 1.2, 7.8 Hz, 1H), 7.16 (d, *J* = 1.2 Hz, 1H), 7.14-7.11 (m, 2H), 4.70 (brs, 1H), 4.39

(t, J = 8.4 Hz, 2H), 3.96 (s, 3H), 3.55-3.45 (m, 4H), 3.41 (s, 3H), 3.40-3.32 (m, 6H), 3.25-3.21 (m, 4H), 2.65 (s, 3H), 2.20-1.95 (m, 2H), 1.75-1.65 (m, 2H).

^{13}C NMR (100 MHz, DMSO-d6) δ 169.2, 166.7, 159.3, 158.7, 158.3, 155.3, 151.4, 148.5, 147.6, 133.2, 130.3, 129.4, 123.0, 119.9, 119.4, 119.2, 118.7, 110.3, 61.5, 58.5, 56.7, 47.6, 46.4, 43.8, 43.2, 41.8, 38.9, 26.8. MS (ESI) *m/z* 583 $(M+H)^+$. HRMS (ESI) *m/z* calc. 583.3145, measured 583.3143 $(M+H)^+$.

Compound **26** was synthesized by the same procedures as the preparation of compound **25**.

11-cyclopentyl-2-((2-ethoxy-4-(4-(4-methylpiperazin-1-yl)piperidine-1-carbonyl)-phenyl)amino)-5-methyl-5H-benzo[e]pyrimido[5,4-b][1,4]diazepin-6(11H)-one (26)

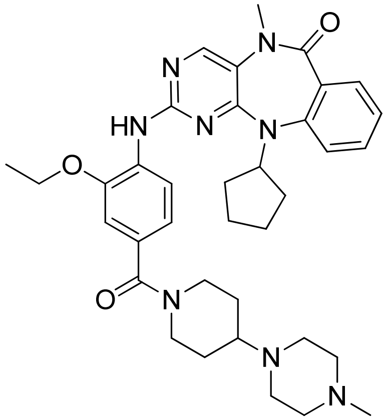

^{1}H NMR (600 MHz, CD_3OD) δ 8.49 (d, J = 8.4 Hz, 1H), 8.36 (s, 1H), 7.64 (dd, J = 1.8, 7.8 Hz, 1H), 7.48-7.45 (m, 1H), 7.27 (d, J = 8.4 Hz, 1H), 7.18 (t, J = 7.8 Hz, 1H), 7.08-7.07 (m, 2H), 4.82-4.78 (m, 1H), 4.19 (q, J = 7.2 Hz, 2H), 3.55-3.48 (m, 6H), 3.45-3.30 (m, 9H), 3.29-3.25 (m, 2H), 2.93 (s, 3H), 2.36-2.33 (m, 1H), 2.15-2.10 (m, 3H), 1.70-1.57 (m, 6H), 1.56-1.54 (m, 1H), 1.47 (t, J = 7.2 Hz, 3H).

^{13}C NMR (100 MHz, DMSO-d6) δ 169.3, 167.7, 164.2, 158.7, 156.1, 152.6, 149.6, 147.8, 132.3, 131.2, 130.3, 128.7, 124.5, 123.7, 121.6, 119.7, 118.9, 110.9, 64.7, 61.3, 57.1, 52.6, 46.3, 42.5, 37.2, 33.1, 32.2, 24.7, 24.5, 15.1. MS (ESI) *m/z* 639 $(M+H)^+$. HRMS (ESI) *m/z* calc. 639.3771, measured 639.3770 $(M+H)^+$.

2. Scatter plot of the cellular results and the enzymatic results against MAPK7 (Fig. S1). Each dot represent the compound, the number correlate with the compound ID. The coordinate of X-axis means the *in vitro* enzymatic IC_{50} of ERK5, and the coordinate of Y-axis means the cellular EC_{50} value for inhibiting epidermal growth factor induced ERK5 autophosphorylation.

a)

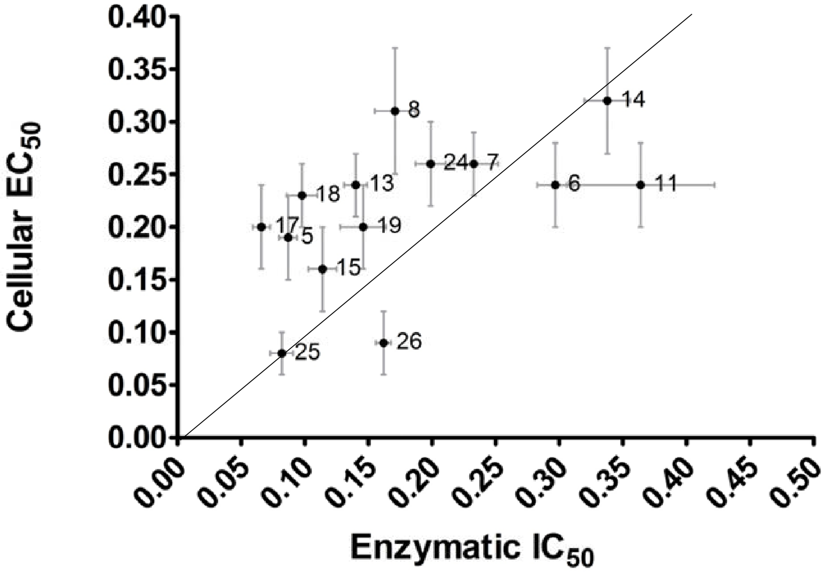

b)

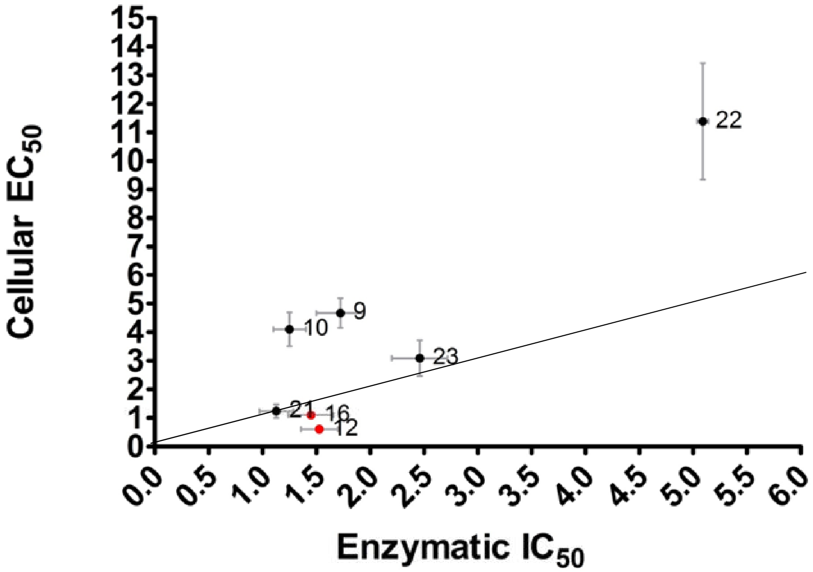

References

[1] Deng, X.; Yang, Q.; Kwiatkowski, N.; Sim, T.; McDermott, U.; Settleman, J. E.; Lee, J. D.; Gray, N. S. Discovery of a benzo[e]pyrimido-[5,4-b][1,4]diazepin-6(11H)-one as a Potent and Selective Inhibitor of Big MAP Kinase 1. *ACS Med. Chem. Lett.* **2011**, *2*, 195-200.

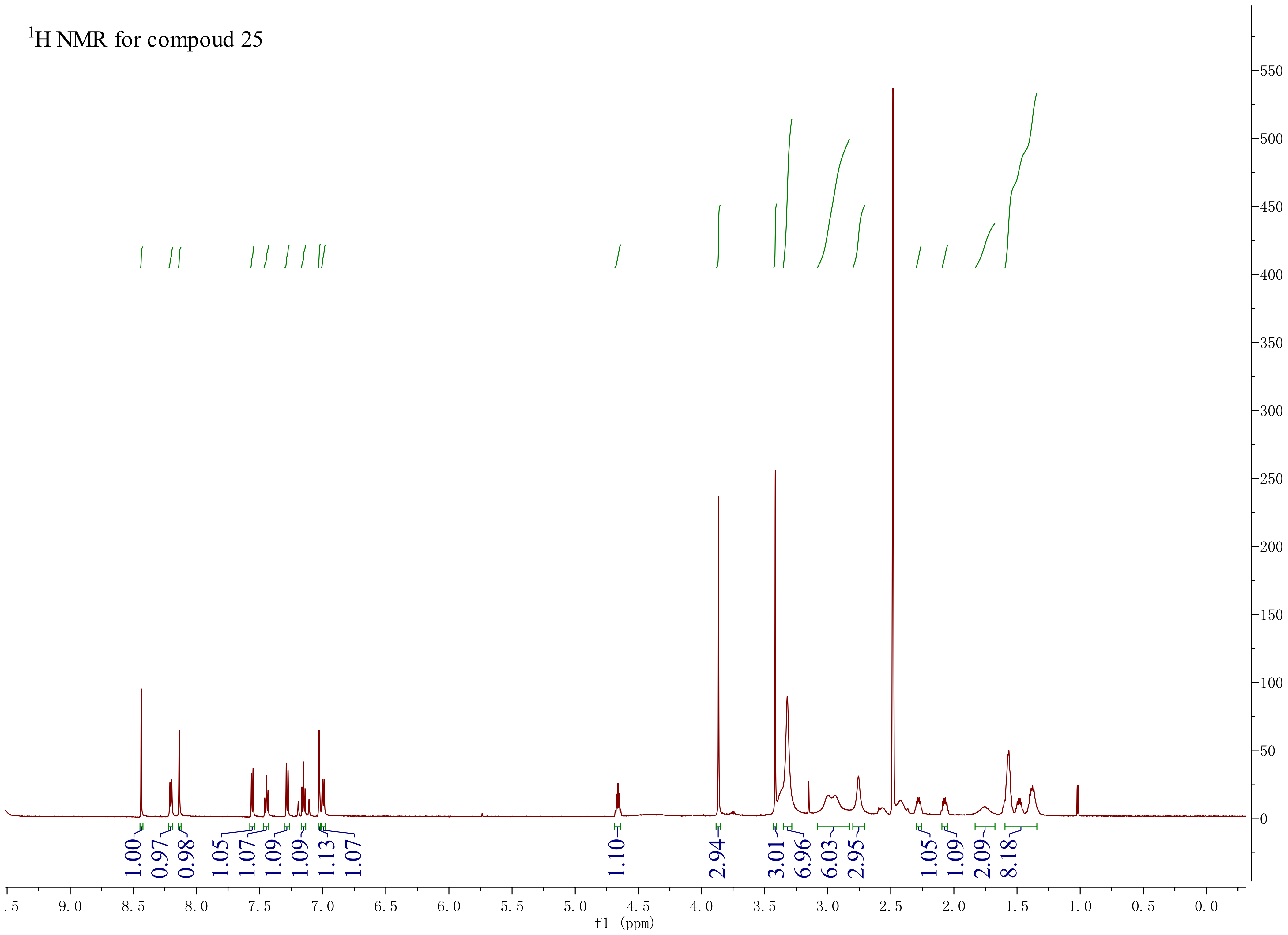

1H NMR for compound 25
1.00
0.97
0.98
1.05
1.07
1.09
1.09
1.13
1.07
1.10
2.94
3.01
6.96
6.03
2.95
1.05
1.09
2.09
8.18
f1 (ppm)

XMD17-26

exp1 CARBON

Compound 25

SAMPLE		PRESATURATION	
date	Mar 26 2013	satmode	n
solvent	dmso	wet	n
file /home/walkup1~		SPECIAL	
/vnmrsys/data/wang~		temp	25.0
5/XMD17-26_2013032~		gain	30
6_01/XMD17-26_2013~		spin	not used
0327_CARBON_01.fid		hst	0.008
ACQUISITION		pw90	6.800
sw	25510.2	alfa	10.000
at	1.285	FLAGS	
np	65536	il	n
fb	17000	in	n
bs	64	dp	y
d1	1.000	hs	nn
nt	10500	PROCESSING	
ct	10500	lb	0.50
TRANSMITTER		fn	not used
tn	C13	DISPLAY	
sfrq	100.498	sp	-1700.8
tof	1530.1	wp	25509.4
tpwr	60	rfl	1701.6
pw	3.400	rfp	0
DECOUPLER		rp	-116.0
dn	H1	lp	0
dof	0	PLOT	
dm	yyy	wc	250
decwave	w	sc	0
dpwr	38	vs	960391
dmf	10020	th	6
		ai	ph

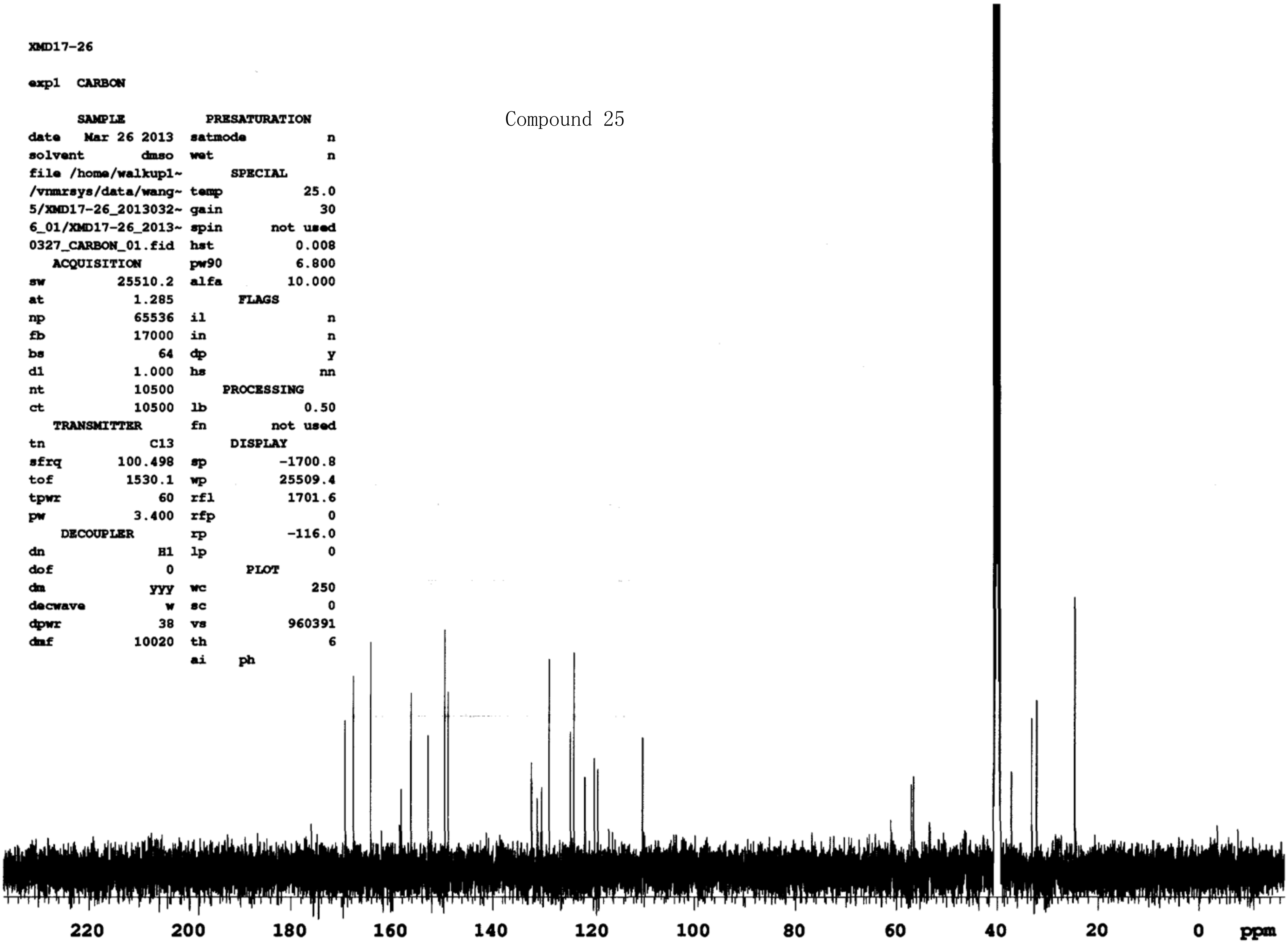

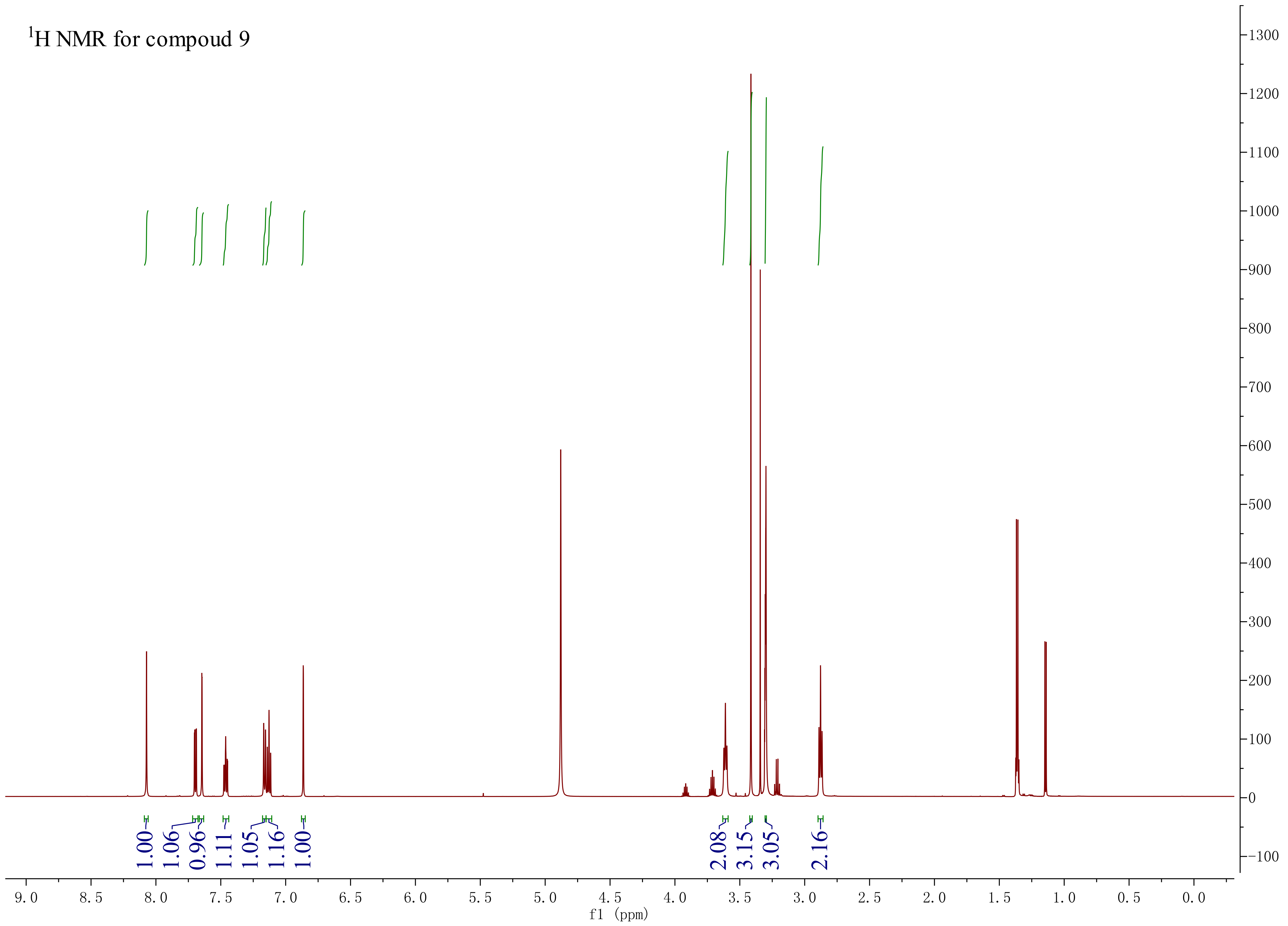

^{1}H NMR for compoud 9
1.00
1.06
0.96
1.11
1.05
1.16
1.00
2.08
3.15
3.05
2.16
f1 (ppm)

XMD17-88

Compound 9

exp1 CARBON

SAMPLE		PRESATURATION	
date	Mar 26 2013	satmode	n
solvent	dmso	wet	n
file /home/walkup1~		SPECIAL	
/vnmrsys/data/wang~		temp	25.0
5/XMD17-88_2013032~		gain	30
6_01/XMD17-88_2013~		spin	not used
0326_CARBON_01.fid		hst	0.008
ACQUISITION		pw90	6.800
sw	25510.2	alfa	10.000
at	1.285	FLAGS	
np	65536	il	n
fb	17000	in	n
bs	64	dp	y
d1	1.000	hs	nn
nt	10500	PROCESSING	
ct	10500	lb	0.50
TRANSMITTER		fn	not used
tn	C13	DISPLAY	
sfrq	100.498	sp	-1700.8
tof	1530.1	wp	25509.4
tpwr	60	rfl	1701.6
pw	3.400	rfp	0
DECOUPLER		rp	-138.7
dn	H1	lp	78.5
dof	0	PLOT	
dm	yyy	wc	250
decwave	w	sc	0
dpwr	38	vs	742948
dmf	10020	th	26
		ai	ph

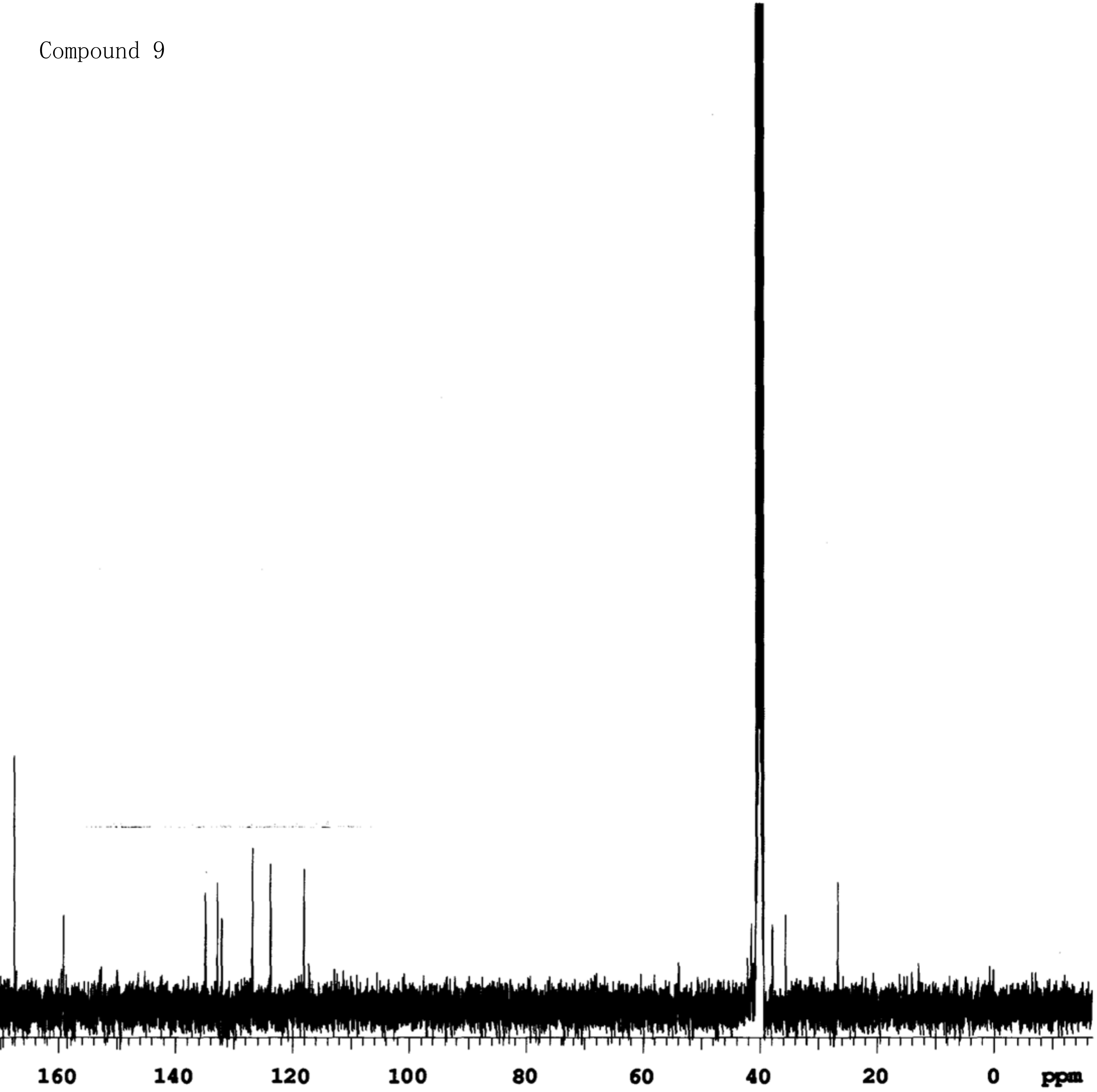

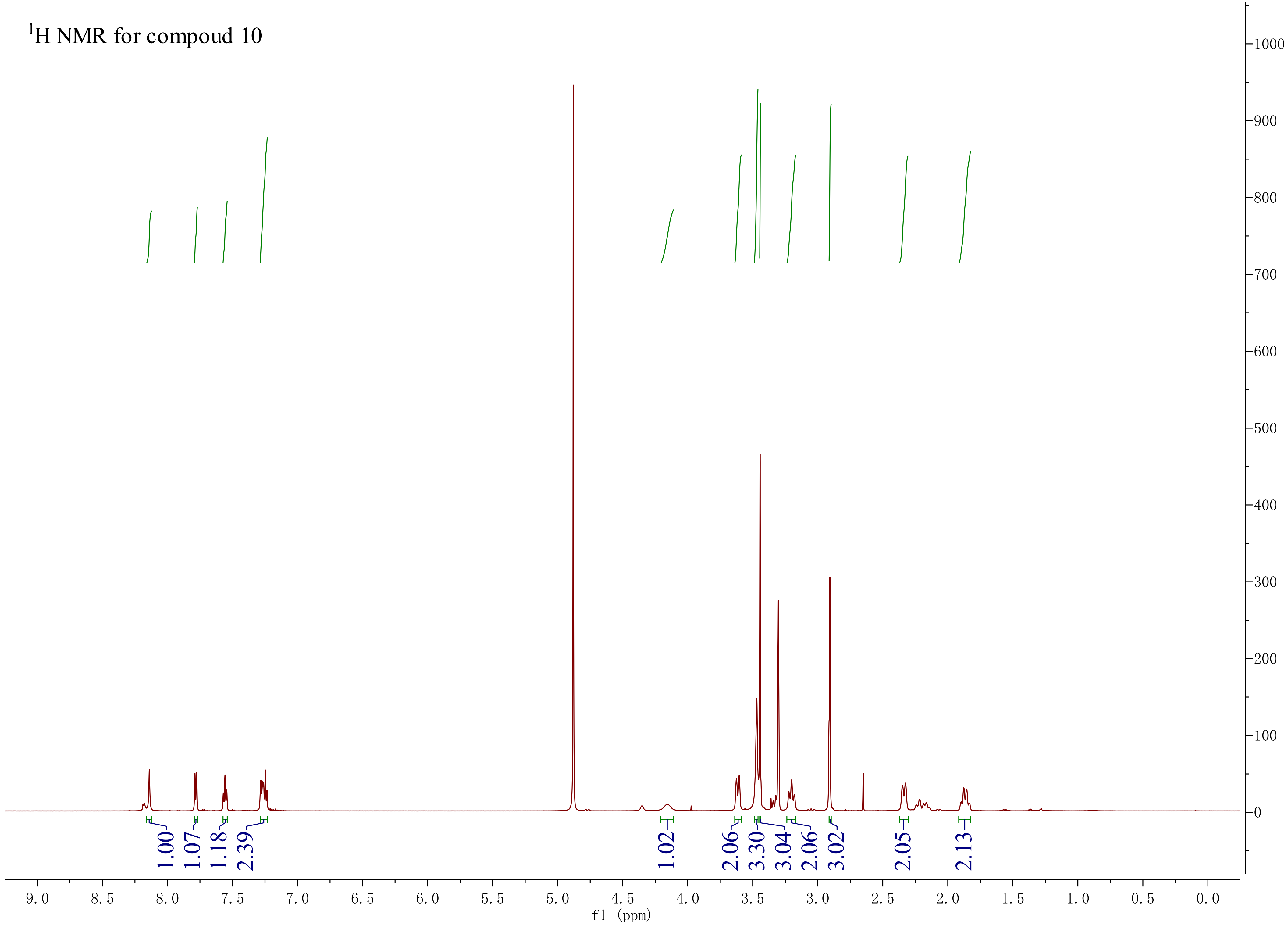

^{1}H NMR for compoud 10
1.00
1.07
1.18
2.39
1.02
2.06
3.30
3.04
2.06
3.02
2.05
2.13
f1 (ppm)

Compound 10

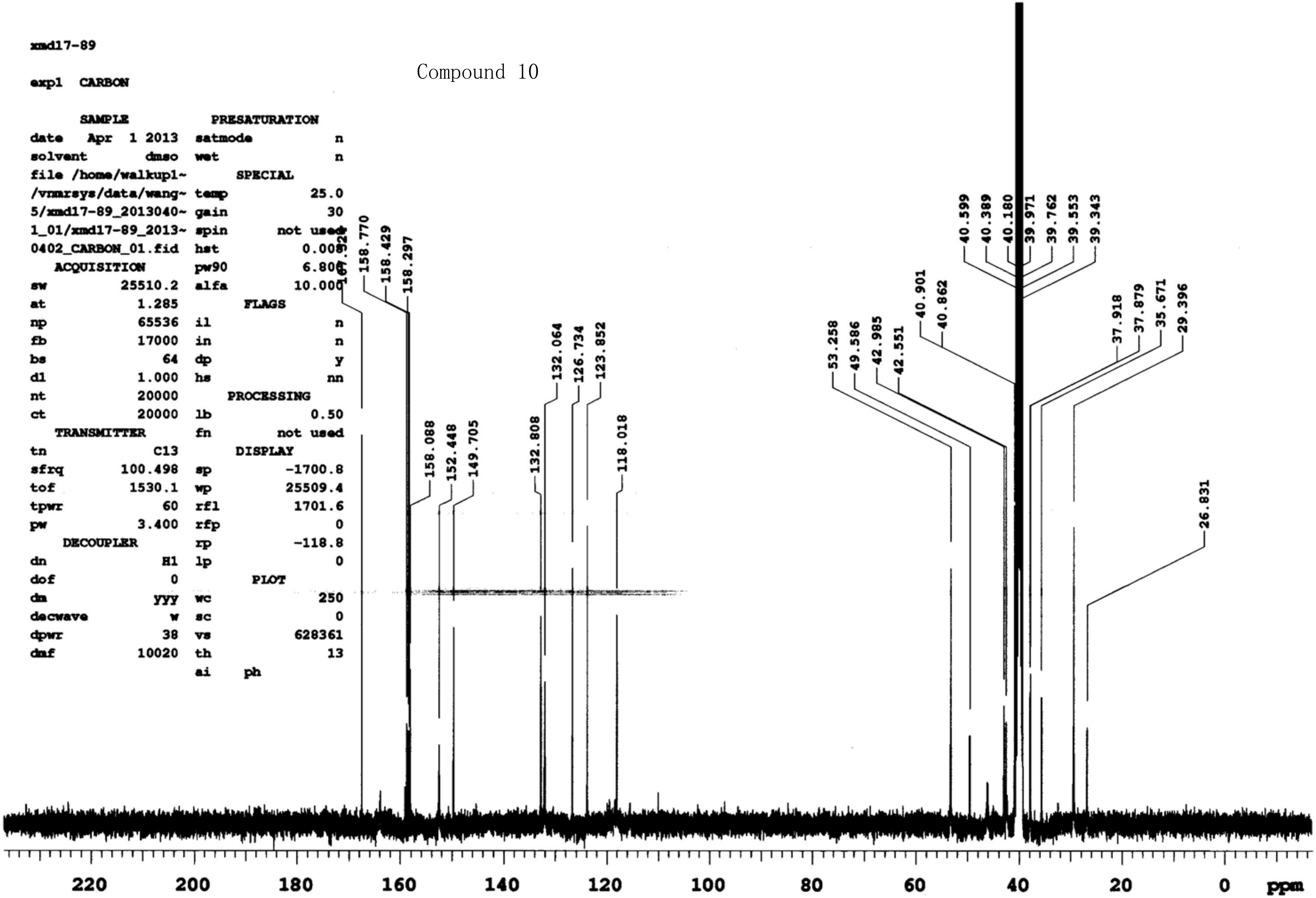
xmd17-89
exp1 CARBON
SAMPLE
date Apr 1 2013
solvent dmso
file /home/walkup1~
/vnmrsys/data/wang~
5/xmd17-89_2013040~
1_01/xmd17-89_2013~
0402_CARBON_01.fid
ACQUISITION
sw 25510.2
at 1.285
np 65536
fb 17000
bs 64
d1 1.000
nt 20000
ct 20000
TRANSMITTER
tn C13
sfrq 100.498
tof 1530.1
tpwr 60
pw 3.400
DECOUPLER
dn H1
dof 0
dm yyy
decwave w
dpwr 38
dmf 10020
PRESATURATION
satmode n
wet n
SPECIAL
temp 25.0
gain 30
spin not used
hst 0.008
pw90 6.800
alfa 10.000
FLAGS
il n
in n
dp y
hs nn
PROCESSING
lb 0.50
fn not used
DISPLAY
sp -1700.8
wp 25509.4
rfl 1701.6
rfp 0
rp -118.8
lp 0
PLOT
wc 250
sc 0
vs 628361
th 13
ai ph
158.770
158.429
158.297
158.088
152.448
149.705
132.808
132.064
126.734
123.852
118.018
53.258
49.586
42.985
42.551
40.901
40.862
40.599
40.389
40.180
39.971
39.762
39.553
39.343
37.918
37.879
35.671
29.396
26.831
220
200
180
160
140
120
100
80
60
40
20
0
ppm

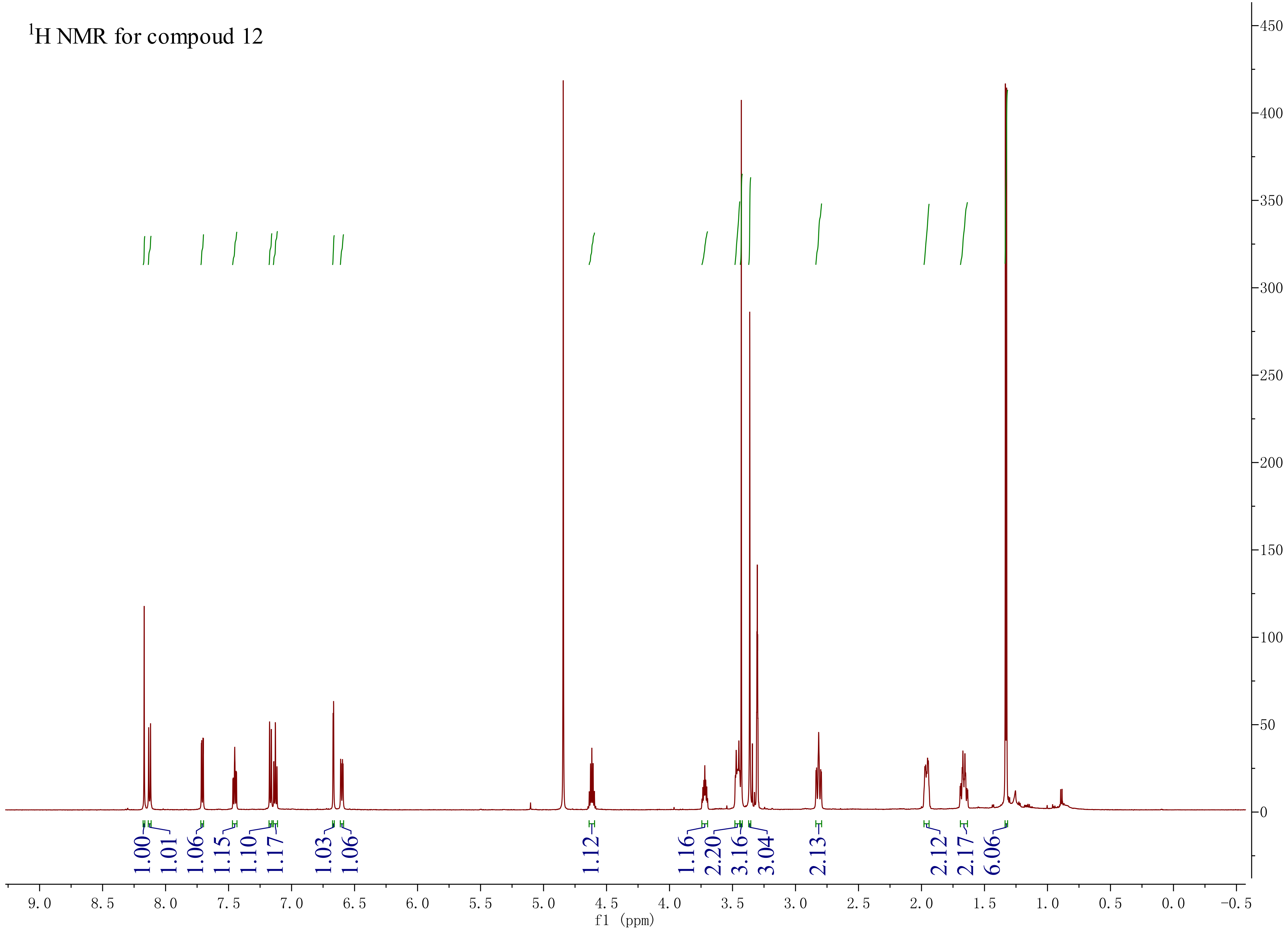

¹H NMR for compoud 12
1.00
1.01
1.06
1.15
1.10
1.17
1.03
1.06
1.12
1.16
2.20
3.16
3.04
2.13
2.12
2.17
6.06
f1 (ppm)

XMD8-86

exp1 CARBON

Compound 12

SAMPLE		PRESATURATION	
date	Mar 25 2013	satmode	n
solvent	dmso	wet	n
file /home/walkup1~		SPECIAL	
/vnmrsys/data/wang~		temp	25.0
5/XMD8-86_20130325~		gain	30
_01/XMD8-86_201303~		spin	not used
26_CARBON_01.fid		hst	0.008
ACQUISITION		pw90	6.800
sw	25510.2	alfa	10.000
at	1.285	FLAGS	
np	65536	il	n
fb	17000	in	n
bs	64	dp	y
d1	1.000	hs	nn
nt	10950	PROCESSING	
ct	10950	lb	0.50
TRANSMITTER		fn	not used
tn	C13	DISPLAY	
sfrq	100.498	sp	-1700.8
tof	1530.1	wp	25509.4
tpwr	60	rfl	1701.6
pw	3.400	rfp	0
DECOUPLER		rp	-120.4
dn	H1	lp	0
dof	0	PLOT	
dm	yyy	wc	250
decwave	w	sc	0
dpwr	38	vs	450287
dmf	10020	th	20
		ai	ph

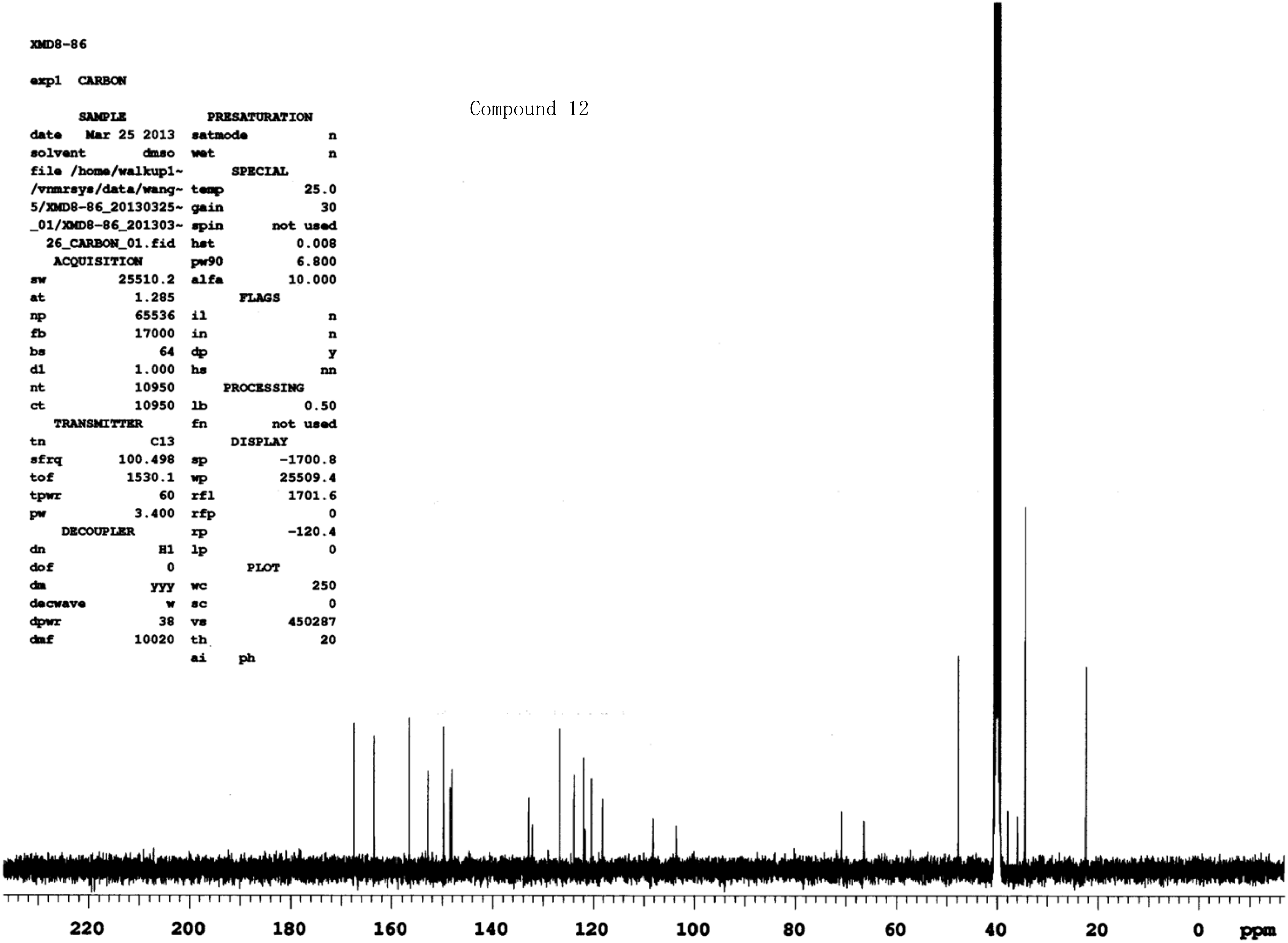

^{1}H NMR for compoud 24

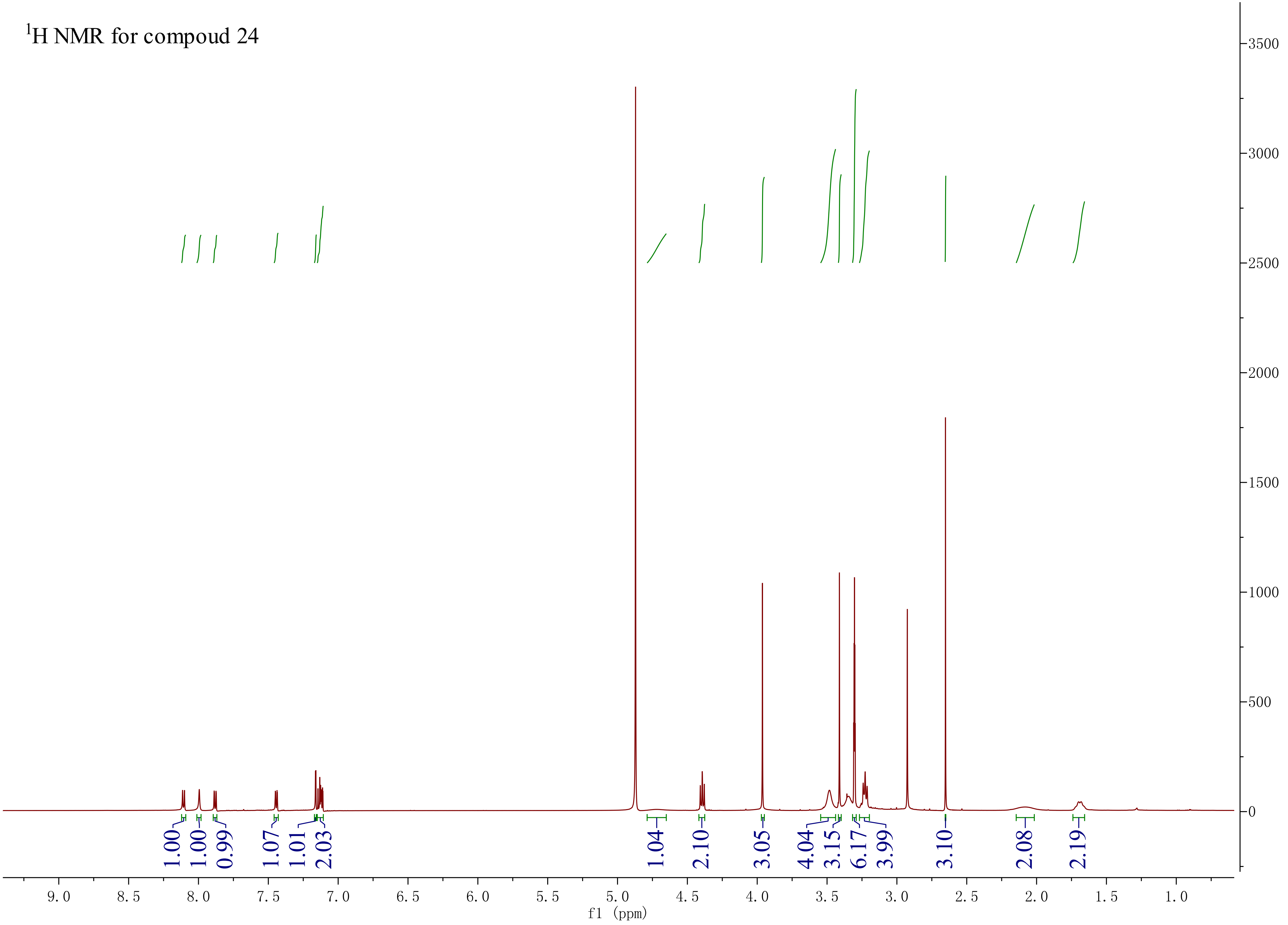

XMD16-44

exp1 CARBON

Compound 24

SAMPLE		PRESATURATION	
date	Mar 27 2013	satmode	n
solvent	dmso	wet	n
file /home/walkup1~		SPECIAL	
/vnmrsys/data/wang~		temp	25.0
5/XMD16-44_2013032~		gain	30
7_01/XMD16-44_2013~		spin	not used
0328_CARBON_01.fid		hst	0.008
ACQUISITION		pw90	6.800
sw	25510.2	alfa	10.000
at	1.285	FLAGS	
np	65536	il	n
fb	17000	in	n
bs	64	dp	y
d1	1.000	hs	nn
nt	21000	PROCESSING	
ct	21000	lb	0.50
TRANSMITTER		fn	not used
tn	C13	DISPLAY	
sfrq	100.498	sp	-1700.8
tof	1530.1	wp	25509.4
tpwr	60	rfl	1701.6
pw	3.400	rfp	0
DECOUPLER		rp	-116.4
dn	H1	lp	0
dof	0	PLOT	
dm	yyy	wc	250
decwave	W	sc	0
dpwr	38	vs	1.30586e+06
dmf	10020	th	19
		ai	ph

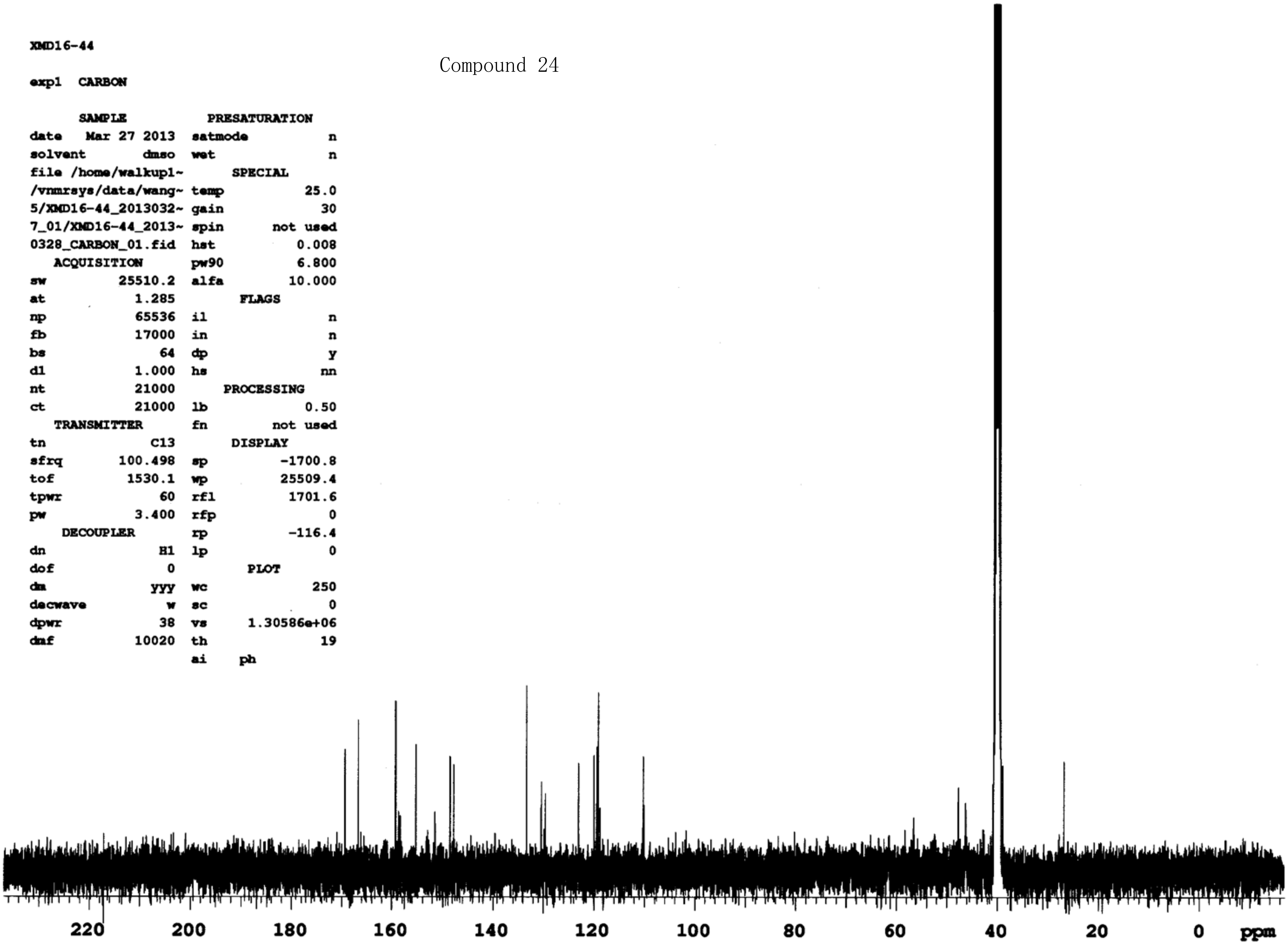

^{1}H NMR for compoud 26

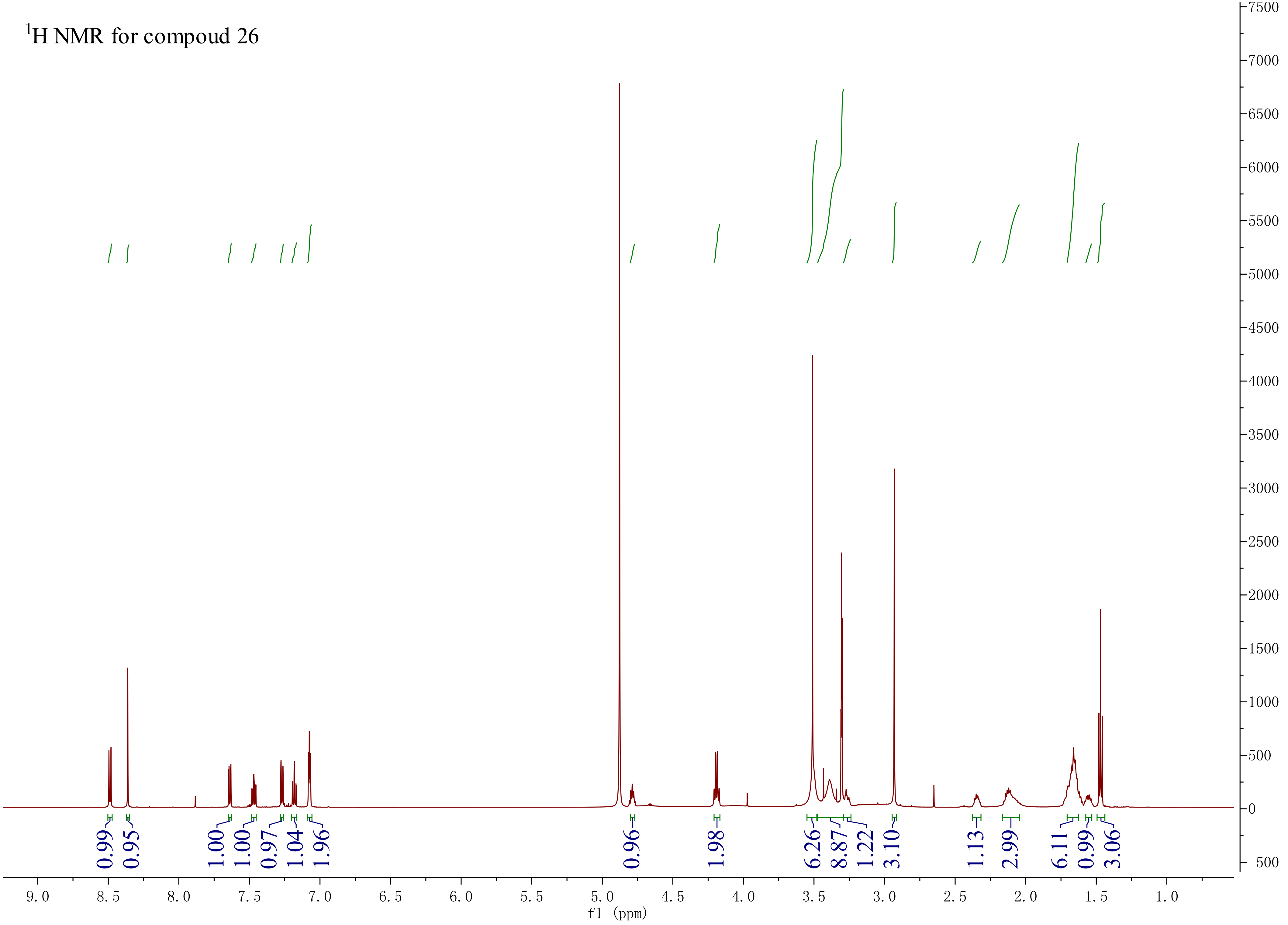

xmd17-109

exp1 CARBON

Compound 26

SAMPLE		PRESATURATION	
date	Mar 27 2013	satmode	n
solvent	dmso	wet	n
file /home/walkup1~		SPECIAL	
/vnmrsys/data/wang~		temp	25.0
5/xmd17-109_201303~		gain	30
27_01/xmd17-109_20~		spin	not used
130327_CARBON_01.f~		hst	0.008
	id	pw90	6.800
ACQUISITION		alfa	10.000
sw	25510.2	FLAGS	
at	1.285	il	n
np	65536	in	n
fb	17000	dp	y
bs	64	hs	nn
d1	1.000	PROCESSING	
nt	10500	lb	0.50
ct	10500	fn	not used
TRANSMITTER		DISPLAY	
tn	C13	sp	-1700.8
sfrq	100.498	wp	25509.4
tof	1530.1	rfl	1701.6
tpwr	60	rfp	0
pw	3.400	rp	-116.7
DECOUPLER		lp	0
dn	H1	PLOT	
dof	0	wc	250
dm	yyy	sc	0
decwave	w	vs	927209
dpwr	38	th	8
dmf	10020	ai	ph

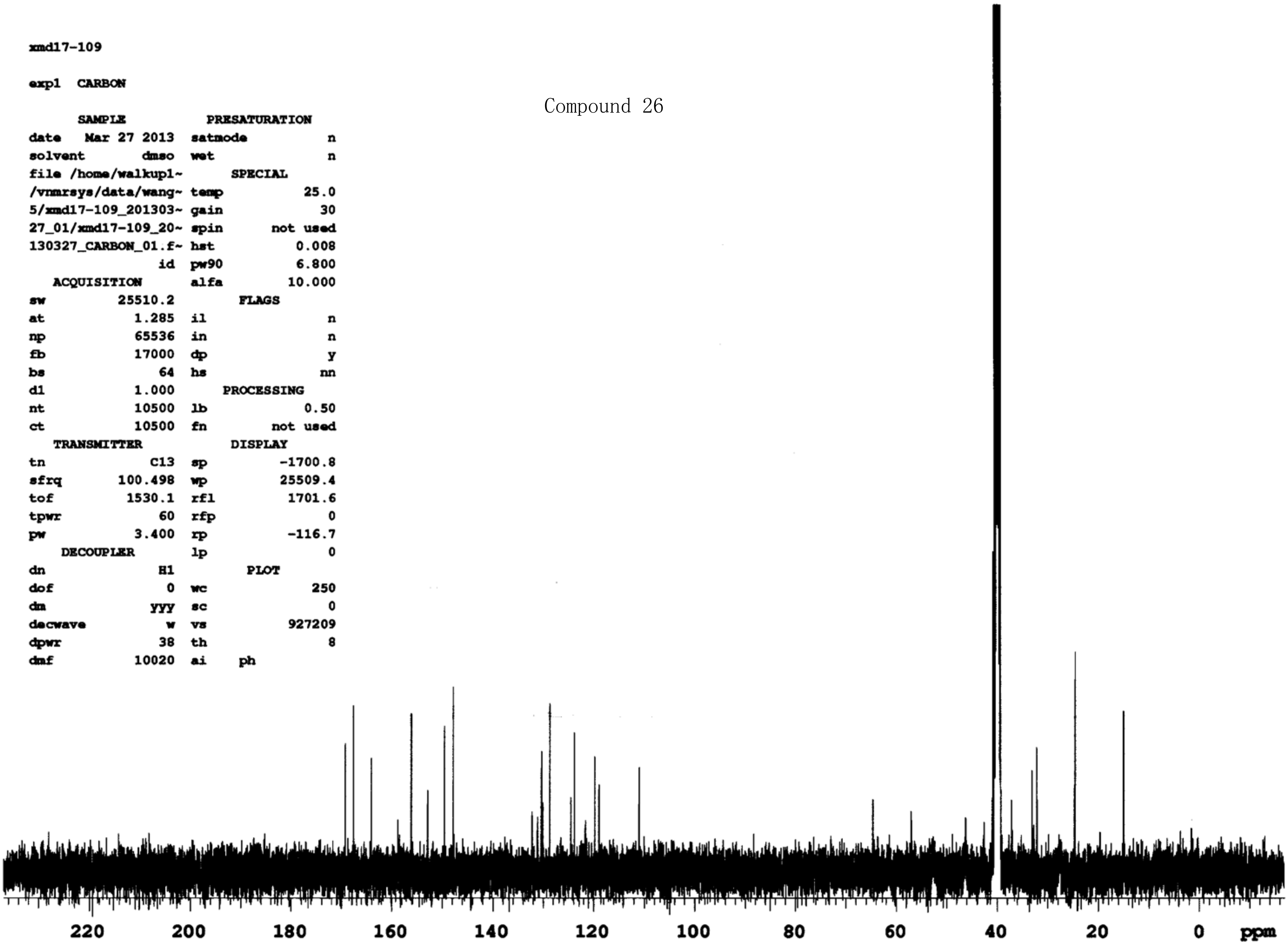

Supplement: Supplementary file 1 [file mmc1.pdf]
